# Genetic Mechanisms in *Apc*-Mediated Mammary Tumorigenesis

**DOI:** 10.1371/journal.pgen.1000367

**Published:** 2009-02-06

**Authors:** Mari Kuraguchi, Nana Yaw Ohene-Baah, Dmitriy Sonkin, Roderick Terry Bronson, Raju Kucherlapati

**Affiliations:** 1 Harvard-Partners Center for Genetics and Genomics, Harvard Medical School, Boston, Massachusetts, United States of America; 2 Division of Genetics, Department of Medicine, Brigham and Women's Hospital, Harvard Medical School, Boston, Massachusetts, United States of America; 3 Rodent Histopathology Core, Dana-Farber Harvard Cancer Center, Boston, Massachusetts, United States of America; University of California Irvine, United States of America

## Abstract

Many components of Wnt/β-catenin signaling pathway also play critical roles in mammary tumor development, yet the role of the tumor suppressor gene *APC* (adenomatous polyposis coli) in breast oncongenesis is unclear. To better understand the role of *Apc* in mammary tumorigenesis, we introduced conditional *Apc* mutations specifically into two different mammary epithelial populations using *K14-cre* and WAP-*cre* transgenic mice that express Cre-recombinase in mammary progenitor cells and lactating luminal cells, respectively. Only the *K14-cre*–mediated *Apc* heterozygosity developed mammary adenocarcinomas demonstrating histological heterogeneity, suggesting the multilineage progenitor cell origin of these tumors. These tumors harbored truncation mutation in a defined region in the remaining wild-type allele of *Apc* that would retain some down-regulating activity of β-catenin signaling. Activating mutations at codons 12 and 61 of either *H-Ras* or *K-Ras* were also found in a subset of these tumors. Expression profiles of acinar-type mammary tumors from *K14-cre*; *Apc^CKO/+^* mice showed luminal epithelial gene expression pattern, and clustering analysis demonstrated more correlation to *MMTV-neu* model than to *MMTV-Wnt1*. In contrast, neither WAP-*cre*–induced *Apc* heterozygous nor homozygous mutations resulted in predisposition to mammary tumorigenesis, although WAP-*cre*–mediated *Apc* deficiency resulted in severe squamous metaplasia of mammary glands. Collectively, our results suggest that not only the epithelial origin but also a certain *Apc* mutations are selected to achieve a specific level of β-catenin signaling optimal for mammary tumor development and explain partially the colon- but not mammary-specific tumor development in patients that carry germline mutations in *APC*.

## Author Summary

Breast cancer is one of the most common malignanices in women in Western countries. Many components of Wnt/β-catenin signaling pathway are known to play critical roles in mammary tumor development, yet the role of the tumor suppressor gene *APC* (adenomatous polyposis coli) in breast oncongenesis is unclear. To study the role of Apc in mammary tumorigenesis, we introduced conditional *Apc* mutations specifically into two different mammary epithelial populations using *K14* (*Keratin 14*)-*cre* and WAP (Whey Acidic Protein)-*cre* transgenic mice that express Cre recombinase in mammary progenitor cells and lactating luminal cells, respectively. In this study, we show that a specific type of *Apc* somatic mutations in mammary progenitor/stem cell population in mice induces mammary carcinomas with histological and molecular heterogeneity, but a complete deletion leads to squamous metaplasia. Our data show that mutations in a multilineage progenitor cell are important in certain mammary tumors. We also show that certain *Apc* mutations are selected to achieve a specific level of β-catenin signaling optimal for mammary tumor development and explain partially the reason why breast cancers do not develop as frequently as colorectal tumors in patients that carry germline mutations in *APC*.

## Introduction

Breast cancer is one of the most common malignancies in women in Western countries and it is the cause of death in approximately 20% of all females who die from cancer. Breast epithelium is a dynamic organ capable of rapid proliferation and functional differentiation upon pregnancy and lactation, followed by involution and remodeling at the end of each lactation period. The adult mammary gland consists of secretory alveoli organized into lobules and interconnected by a system of branching ducts. The entire mammary epithelium is enveloped by a basement membrane and embedded in a fatty connective tissue called the mammary fat pad. In the ducts and alveoli, the mammary epithelium is organized into two layers, a basal layer of myoepithelial cells and a luminal epithelial layer. The myoepithelial cells, like other basal epithelial cells, express basal keratins (in particular, K5 and K14), P-cadherin, and the transcription factor p63 [1]. They also contain smooth muscle-specific proteins, including the α-smooth muscle actin (α-SMA), which confer contractility. By contrast, luminal cells express K8 and K18, which are characteristics of simple epithelia and when fully differentiated, secrete milk proteins [1].

The molecular mechanisms of the initiation of breast cancer are well studied. Mutations in *BRCA1* and *BRCA2* result in increased susceptibility to breast cancer [2] and mutations in *TP53* are found to be common in late stages of this cancer [3]. It has been shown that dysregulation of the Wnt signaling pathway is an important contributor to the initiation of breast cancer [4]. *Adenomatous Polyposis Coli* (*APC*) is a member of the Wnt/β-catenin signaling pathway that is involved in the maintenance of the progenitor cell population in the skin, intestine and other tissues. Mutations and/or altered expression in the tumor suppressor gene *APC* are frequently found in sporadic breast cancers [5]–[7] which implicates its role as a tumor suppressor in mammary epithelium. In mouse, activation of Wnt/β-catenin signaling in the mammary epithelium either by mutation in *Apc* (GenBank NM_007462) or by stabilization of β-catenin (NM_007614), contributes to tumorigenesis. For example, mice heterozygous for germline mutation in *Apc* (*Apc^Min^*) spontaneously develop mammary tumors, although at a significantly lower incidence than intestinal tumors [8]. Transient expression of an activated form of β-catenin in secretory luminal epithelium driven by the MMTV promoter leads to both mammary gland hyperplasia and mammary adenocarcinoma [9]. Similarly, expression of a transcriptionally active form of β-catenin lacking the N-terminal 89 amino acids (ΔN89 β-catenin) results in precocious development, differentiation, and neoplasia in both male and female mouse mammary glands [10]. The *K5* promoter-driven expression of stabilized N-terminally truncated β-catenin (ΔN57 β-catenin) in the basal epithelial layer of the mammary gland, led to basal-type mammary hyperplasia and invasive carcinomas [11]. Contrasting results have been obtained when Wnt/β-catenin signaling pathway was stably activated constitutively in luminal cells of mammary epithelium using Cre-loxP technology. The stabilization of β-catenin, aided either by Cre-mediated oncogenic activation of β-catenin or Apc deficiency, induced transdifferentiation into epidermis and squamous metaplasia of the mammary epithelium but failed to induce neoplasia [12],[13]. Apc deficient luminal epithelium developed acanthomas only in the additional absence of Tcf-1 [13]. Together, these results indicate a key role for Apc in both mammary gland development and tumorigenesis, most likely through activation of β-catenin signaling, but it is still unclear why the variation in methods of β-catenin signaling activation can produce different phenotypes in mammary glands. These results suggest that the timing and the cell types in which the *Apc* mutations occur might be important for breast cancer development.

To better understand how Apc inactivation in the mammary epithelium results in cancer, we crossed mice carrying a floxed allele of *Apc* to *K14*-cre and whey acid protein (WAP)-*cre* transgenic mice. *K14*-expression starts embryonically in cells that give rise to both basal and luminal cells of mammary gland, while WAP expression is restricted to adult females following pregnancy and lactation. We show here that *K14*-mediated *Apc* heterozygosity directly resulted in mammary adenocarcinoma development, but WAP-mediated *Apc* deficiency resulted in severe squamous metaplasia and not readily in neoplasia. The expression of both luminal and myoepithelial lineage markers, as well as the presence of the common initiating somatic *Apc* mutation in histologically distinct regions of a tumor, is in line with the progenitor cell origin of *K14-cre*; *Apc^CKO/+^* tumors. The remaining wild-type allele of *Apc* in these tumors harbored truncation mutation in a specific region of the gene, which seems to be selected for mammary tumorigenesis. These results show that the timing and cell type in which the critical mutational events occur and the level of resultant activation of the β-catenin signaling cascade are critical for the initiation of mammary tumor development.

## Results

### Mammary Tumor Susceptibility in *K14-Cre*; *Apc^CKO/+^* Mice but Not in WAP-Cre–Induced *Apc* Mutant Mice

**Figure 1:**
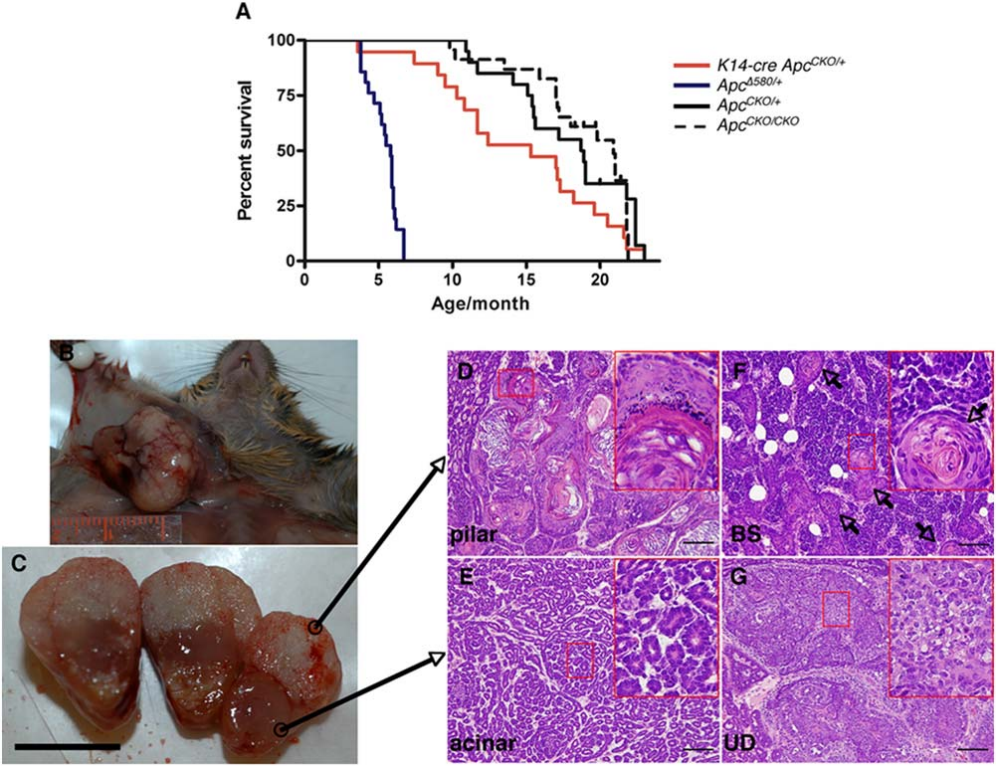
Mammary tumorigenesis in *K14-cre*; *Apc^CKO/+^* female mice. (A) Survival Curve of *K14-cre Apc^CKO/+^* mice. Kaplan-Meier survival plot of *K14-cre*; *Apc^CKO/+^* (red solid line, n = 19), *Apc^Δ580/+^* (blue solid line, n = 21), *Apc^CKO/CKO^* (broken line, n = 37) and *Apc^CKO/+^* mice (solid line, n = 33). Germline *Apc* heterozygous (*Apc^Δ580/+^*) mice are predisposed to multiple intestinal tumorigenesis, but *K14-cre* driven *Apc* heterozygosity have decreased survival compared to the controls primarily due to predisposition to mammary tumors. (B, C) A mammary tumor (C) from a *K14-cre*; *Apc^CKO/+^* female mouse (B) with grossly biphasic growth. Scale bar 1 cm. (D–G) *K14-cre*; *Apc^CKO/+^* tumors show a spectrum of histological patterns from differentiated pilar (D), acinar (E), basosquamous (BS, F) to undifferentiated (UD, G), usually in combinations of these. Squamous metaplasia (arrows) is frequent in these tumors, showing transdifferentiation to epidermal and pilar structures. Insets are the area within respective red boxes at higher magnification. Scale bars 100 µm.

*Apc^Δ580/+^* mice, a germline knockout strain derived from the *Apc* conditional mice die primarily due to development of multiple intestinal tumors [14]. We have found that these mice can occasionally develop mammary tumors, as in *Apc^Min/+^* mice [8] although at a low incidence (14.3%, 3 of 21). To further study the role of Apc in mammary tumor development without being hindered by the intestinal tumorigenesis, we induced *Apc* mutations specifically in mammary epithelium using either *K14* or WAP promoters. We have previously shown that homozygous loss of *Apc* in *K14*-expressing embryonic cells results in abnormal skin phenotype associated with aberrant development and squamous metaplasia in many epithelial-derived tissues including teeth and thymus, and die prior to weaning [14]. In contrast to *K14-cre*; *Apc^CKO/CKO^* mice, the *K14-cre*; *Apc^CKO/+^* mice were phenotypically normal at birth, but upon aging showed decreased survival primarily due to mammary tumor susceptibility in female mice (Table 1, Figure 1A–G). The *K14-cre*; *Apc^CKO/+^* female mice (n = 19) had a median survival of 15-months. We were able to carefully analyze 17 of these mice for pathology. The differences in survival are statistically significant between the *K14-cre*; *Apc^CKO/+^* female mice and the *cre*-negative *Apc^CKO/+^* and *Apc^CKO/CKO^* female mice (p<0.02, log rank test). A large proportion of *K14-cre*; *Apc^CKO/+^* females invariably developed mammary tumors with focal squamous metaplasia (13 of 17, 76.5%, Figure 1B–G) and the mice were sacrificed when their tumors were over 2 cm in diameter. Of the four mammary tumor-free female mice, three died of hepatoma, histiocytic sarcoma and myxosarcoma, respectively and one succumbed to severe dermatitis. We extensively performed a full histological autopsy on seven mammary tumor-bearing mice, of which two had lung metastasis. The mammary tumor susceptibility was also observed in *K14-cre*; *Apc^CKO/+^* female mice backcrossed to C57BL/6 (*K14-cre*; *Apc^CKO/+^*-B6, n = 8). At the time of analysis, half of them (4 of 8, 50%) developed mammary tumors before reaching 12-months of age while the other half remained tumor-free for over 12-months.

**Table 1:** Incidence of tumorigenesis in *Apc* mutant female mice.

Genotype		Parity	Age	Median Survival Age/mo	N	Mammary tumors % (n)	Intestinal tumors % (n)	Other % (n)	Malignancy % (n)
Cre	Apc								
K14-cre	CKO/+	null	15.2±4.8	15.3	17	76.5 (13)	0 (0)	23.5 (4)	94.1 (16)
WAP-cre	CKO/+	multi	18.3±2.5	18.9	6	16.7 (1)	0 (0)	0 (0)	16.7 (1)
WAP-cre	CKO/CKO	multi	18.0±2.7	19.2	14	14.3 (2)	0 (0)	21.4 (3)	35.7 (5)
WAP-cre	CKO/+	null	17.6±4.3	18.7	8	12.5 (1)	0 (0)	25.0 (2)	37.5 (3)
WAP-cre	CKO/CKO	null	17.9±2.9	18.0	10	10.0 (1)	0 (0)	10.0 (1)	20.0 (2)
+	Δ580/+	null	5.4±1.0	5.8	21	14.3 (3)	100 (21)	0 (0)	100 (21)
+	CKO/+	-	18.7±3.7	19.0	33	3.0 (1)	0 (0)	6.0 (2)	9.1 (3)
+	CKO/CKO	-	18.9±4.5	19.6	37	5.4 (2)	0 (0)	8.1 (3)	13.5 (5)

N = total number of mice in the study.

Both WAP-*cre* positive heterozygous and homozygous *Apc^CKO^* mice were born in the expected Mendelian ratio with no bias towards either sex. All mice were phenotypically normal at birth, developed normally and were fertile. However, litters from WAP-*cre;Apc^CKO/CKO^* mothers could not thrive. When litters were transferred to foster mothers, these litters survived and developed normally, suggesting that it was due to lack of appropriate milk production by WAP-*cre;Apc^CKO/CKO^* mothers and consequent inability to nurse their litters properly. This observation is in agreement with the BLG-*cre* mediated inactivation of *Apc* [13]. Both WAP-*cre* positive heterozygous and homozygous *Apc^CKO^* female mice have been allowed to pass through four complete lactation cycles with the exception of the two mice that underwent three and were monitored up until they were 18-months of age. Nulliparous females of the same genotypes were also monitored as their controls. Unlike in the *K14-cre;Apc^CKO/+^* nulliparous female mice that developed mammary tumors spontaneously, neither mated WAP-cre positive *Apc^CKO/CKO^* nor *Apc^CKO/+^* mice showed mammary tumor susceptibility (Table 1). There were hardly any differences in either the survival or tumorigenicity between multiparous and nulliparous WAP-cre positive females of either *Apc^CKO^* genotypes and all of them lived as long as Cre negative controls (Table 1). Mammary tumors were occasionally observed in all four groups of WAP-cre positive females (Table 1). Mammary tumors developed in two out of 14 (21.4%) WAP-*cre;Apc^CKO/CKO^* and one of 6 (16.7%) WAP-*cre;Apc^CKO/+^* multiparous females, whereas those in nulliparous females were one of 10 (10%) and one of eight (12.5%), respectively.

Examination of mammary glands from the aged multiparous WAP-*Cre;Apc^CKO/CKO^* female mice revealed severe squamous metaplasia in mammary glands that explains the inability of these females to produce milk (Figure S1A, B), whereas those of the age matched multiparous WAP-*Cre;Apc^CKO/+^*, nulliparous WAP-*Cre;Apc^CKO/+^* and nulliparous WAP-*Cre;Apc^CKO/CKO^* females had histologically virginal state without any acini development. The extent of metaplasia was so severe in multiparous WAP-*Cre;Apc^CKO/CKO^* mice that almost all acini had squamous metaplasia, some with mineralization, showing osteometaplasia (Figure S1B). This observation is analogous to BLG-*cre*-mediated inactivation of *Apc* [13] and WAP-*cre*-mediated activation of oncogenic β-catenin [12], further supporting that the homozygous mutations of *Apc* in mammary epithelium perturbs normal mammary differentiation and causes transdifferentiation, but does not readily result in tumorigenesis. These results suggest the timing and perhaps the cell type in which the *Apc* mutations occur is critical for mammary tumor development.

### Histological Heterogeneity and the Expression of both Luminal and Myoepithelial Markers in *K14-cre*; *Apc^CKO/+^* Mammary Tumors but Not in WAP-Cre–Induced *Apc* Mammary Tumors

Tumors arising from stem or progenitor cells may show mixed lineage differentiation [15]. *K14*-expression starts embryonically, and some of those cells give rise to both basal and luminal cells of mammary gland [16], while WAP expression starts in adult luminal mammary epithelium following pregnancy and lactation [17]. To investigate whether *Apc* mutation-induced tumors arising from *K14-cre* positive cells and WAP-*cre* positive cells have similar histology and lineage differentiation, these tumors were histologically examined and stained for both K8, a marker for luminal epithelial cells, and α-SMA, K14 and p63, markers for basal myoepithelial cells.

**Figure 2:**
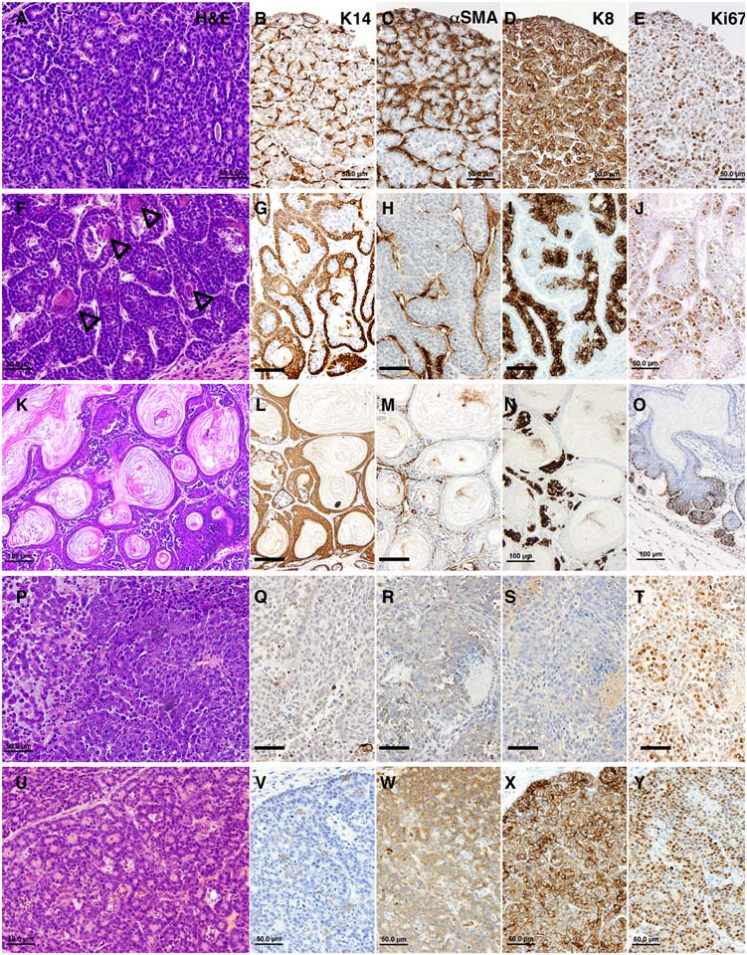
Histological heterogeneity in mammary tumors from *K14-cre*; *Apc^CKO/+^* female mice. (A–E) Acinar tumor. (F–J) Basosquamous tumor with squamous metaplasia and ghost cells indicated by arrowheads. (K–O) Pilar tumor with extensive keratinization. (P–T) Undifferentiated tumor. (U–Y) Mammary tumor from a multiparous WAP-*cre*; *Apc^CKO/CKO^* female mouse. Stained with H&E for histology (A, F, K, P, U), K14 (B, G, L, Q, V), α-SMA (C, H, M, R, W), K8 (D, I, N, S, X) and Ki67 (E, J, O, T, Y) antibodies as indicated at the top. Note the different patterns of cell lineage markers associated with different tumor descriptors. Scale bars are 100 µm for K–O, the rest are 50 µm.

The histology of mammary tumors that developed in the germline knockout strain, *Apc^Δ580/+^* mice were similar to those described for *Apc^Min/+^* mammary tumors [18]. All three of them were pilar tumors with extensive keratinization, and were adjacent to basosquamous components. The mammary tumors developed in *K14-cre*; *Apc^CKO/+^* mice, either in mixed or C57BL/6 background, exhibited a variety of histological patterns within a tumor similar to those found in other Wnt Pathway tumors [18]. Most of the tumors were adenocarcinomas with focal squamous metaplasia (Figure 1B–G, 2). Squamous metaplasia may be extensive as in pilar tumors or scattered as multiple foci. The most common histological pattern observed in 16 *K14-cre*; *Apc^CKO/+^* mammary tumors from mice in the mixed background were acinar (Figure 1E, 2A–E), often associated with basosquamous (Figure 1F, 2F–J) and pilar (Figure 1D, 2K–O) components but only occasionally with undifferentiated component (Figure 1G, 2P–T). Upon backcrossing to C57BL/6, the *K14-cre*; *Apc^CKO/+^* mammary tumors (n = 4) developed were primarily composed of basosquamous and pilar histological types with extensive keratinization, similar to *Apc^Δ580/+^* tumors, and acinar histology was no longer observed. All *K14-cre*; *Apc^CKO/+^* mammary tumors exhibited multiple histological patterns within a tumor, some more prominent than the others. In two cases, the tumors appeared grossly biphasic with distinct keratinized and solid portions (Figure 1B, C). Histologically, the keratinized portion had pilar structures with a number of keratinizing cysts (Figure 1D, 2K), and the solid cellular portion had the acinar pattern (Figure 1E, 2A) when the tumor derived from a mixed background *K14-cre*; *Apc^CKO/+^* mouse. A similar biphasic growth was also observed in two mammary tumors from *K14-cre*; *Apc^CKO/+^*-B6 mice, but the solid portion had basosquamous pattern. Immunochemical examination of these tumors revealed that these tumors are composed of both luminal and myoepithelial cells, with α-SMA and K14 positive myoepithelial cells forming a single layer around the K8-positive tumor cells in a well-organized structure as in normal ducts (Figure 2B–D, G–I). The immunochemical patterns for K14 and K8 shifts to those of epidermis in pilar structures (Figure 2L–N). Only in undifferentiated mammary tumor components, the expression of both lineage markers was lost (Figure 2Q–S). These tumors were highly proliferative as determined by Ki67 staining (Figure 2E, J, O, T), although the proliferation pattern of the pilar tumors was restricted to basal layer, analogous to that of hair follicles. Strong positivity for Tcf/β-catenin target genes, Myc and cyclin D1, demonstrating the activation of the Wnt/β-catenin pathway were also observed in these tumors (Figure S2A–G). Like in many other mouse mammary tumor models, *K14-cre*; *Apc^CKO/+^* mammary tumors were negative for hormone receptors, Estrogen Receptor (ER) and Progesterone Receptor (data not shown).

Most WAP-*cre* induced tumors, of which three were from WAP-*cre*; *Apc^CKO/CKO^* and two were from WAP-*cre*; *Apc^CKO/+^* mice, were histologically acinar or glandular-like (Figure 2U–Y). Interestingly, all except one were only positive for K8 and did not show defined expression of myoepithelial markers, K14 and p63, as in *K14-cre*; *Apc^CKO/+^* mammary tumors, and α-SMA expression was very diffuse and aberrant (Figure 2V–X). They also had squamous metaplasia where K14 expression is observed but with less defined structures than those observed in *K14-cre*; *Apc^CKO/+^* tumors (Figure S1C, D). These observations suggest that *K14-cre*; *Apc^CKO/+^* mammary tumors derived from either stem or progenitor cells of the mammary gland while WAP-*cre* induced tumors derived from more differentiated cells of mammary luminal cells.

### Nature and Location of Second Hit *Apc* Mutations in *K14 cre*; *Apc^CKO/+^* Tumors

**Figure 3:**
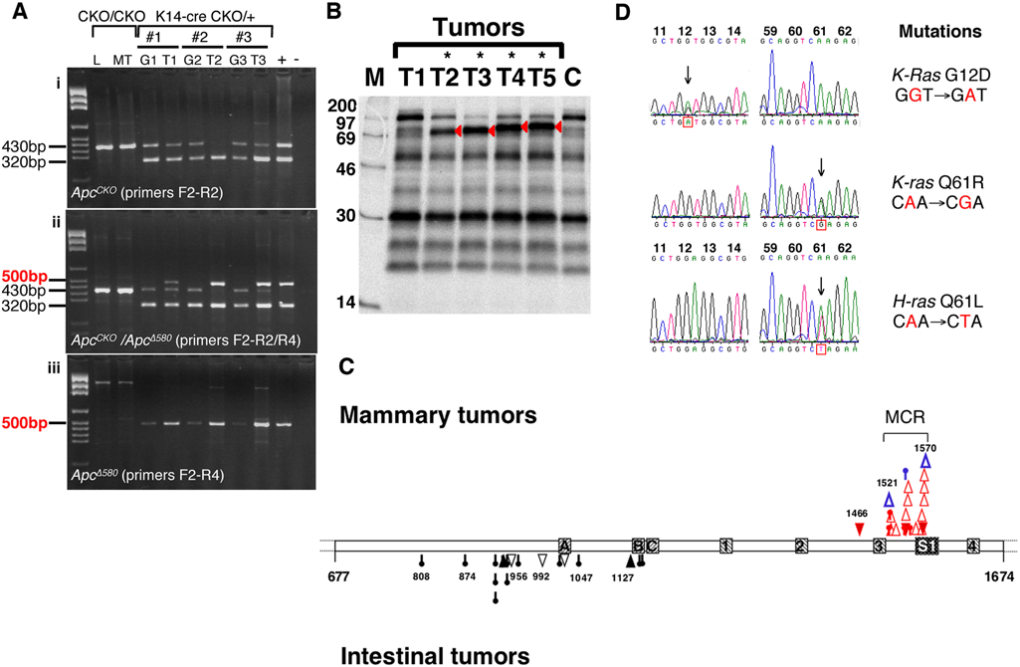
Somatic mutations in mammary tumors from *K14-cre*; *Apc^CKO/+^* mice. (A) Genotyping for *Apc* in *K14-cre*; *Apc^CKO/+^* mammary tumors revealing that the conditional *Apc* allele has been recombined to the *Apc^Δ580^* allele. Genotyping PCR for the (i) wild-type and *Apc^CKO/+^* alleles, (ii) wild-type, *Apc^CKO/+^* and *Apc^Δ580^* alleles, (iii) *Apc^Δ580^* allele alone. The faint presence of the *Apc^Δ580^* allele (500 bp band) in *K14-cre*; *Apc^CKO/+^* mammary glands is only detectable by allele specific PCR (iii), whereas the corresponding mammary tumors shows the prominent presence of the deleted allele over the *Apc^CKO/+^* allele (430 bp band) in 3-allele PCR (ii). Note that the wild-type allele (320 bp band) is still intact in all the mammary tumor samples. Deleted *Apc* products are not detected in either liver or mammary tumors from *K14-cre* negative mice. L = Liver, G = Mammary Gland, MT or T = Mammary Tumor. (B) Wild-type allele-specific *in vitro* transcription and translation assay of *Apc* for detection of truncation mutations. The majority of tumors (19 of 20) showed truncated products (red arrows) within the analyzed region (codons 677–1674) of the remaining wild-type allele of *Apc*. Note that two histologically distinct regions, acinar (T4) and pilar (T5) analyzed individually share the same mutations. (C) Distribution of *Apc* mutations in *K14-cre*; *Apc^CKO/+^* mammary tumors. Diagram of Apc between codons 677 and 1674 showing positions and characteristics of truncation mutations detected in *K14-cre*; *Apc^CKO/+^* (red) and *Apc^Δ580/+^* (blue) mammary tumors (above). The mutation distribution of *Apc^1638N^* intestinal tumors [50] is also shown for comparison (below). Each symbol, deletion (Empty triangle), insertion (filled triangle), substitution (pinhead), represents an independent mutation. Note the mutation cluster region (MCR) of mammary tumors is located further downstream than the region frequently observed in intestinal tumors. The three 15-aa (A,B,C) and four 20-aa (1, 2, 3, 4) β-catenin binding repeats and one SAMP repeat (S1) in this segment of Apc are indicated. (D) Mutations in the *K-ras* and *H-ras* gene. Representative examples of somatic mutations in the *K-Ras* and *H-Ras* cDNA from mammary tumors of the *K14-cre*; *Apc^CKO/+^* mice. Sequence chromatograms of codons 12, 13 and 61 are shown. Mutant peaks are indicated by arrows. Mutations found in either *K-Ras* or *H-Ras* were mutually exclusive.

It is known that in *Apc*-mediated tumorigenesis, an important initial event is the loss or mutation of the second copy of the *Apc* locus. We examined the status of *Apc* in 20 *K14-cre*; *Apc^CKO/+^* (16 mixed, 4 C57BL/6 backgrounds) and five WAP-*cre;Apc^CKO^* (both WAP-*cre;Apc^CKO/+^* and WAP-*cre;Apc^CKO/CKO^*) mammary tumors. We performed 3 separate PCRs to respectively screen for the (i) wild-type (320 bp) and *Apc^CKO/+^* (430 bp) alleles, (ii) wild-type, *Apc^CKO/+^* and *Apc^Δ580^* (500 bp) alleles, and (iii) *Apc^Δ580^* allele alone to genotype tumor DNA as shown in Figure 3A, i–iii. Skin and mammary glands are the tissues known to have transgene expression in *K14-cre* mice [16] and the presence of the deleted allele of *Apc* can be detected in small quantities in normal mammary glands of *K14-cre*; *Apc^CKO/+^* mice only by the *Apc^Δ580^* allele-specific PCR (Figure 3A iii; G1∼G3). In contrast, most *K14-cre*; *Apc^CKO/+^* tumors had *Apc^Δ580/+^* genotype, with no or reduced presence of *Apc^CKO^* allele (Figure 3A i and ii; T1∼T3), showing that these tumors were derived from the clonal expansion of *Apc^Δ580/+^* cells. It is important to note that all *K14-cre*; *Apc^CKO/+^*-derived mammary tumors (20 of 20) were heterozygous for *Apc^Δ580^* mutation but still retained the wild-type allele and did not show allelic loss. In WAP-cre positive *Apc^CKO^* mice, the presence of the *Apc^Δ580^* allele was only detected in multiparous WAP-*cre;Apc^CKO^* mammary glands (Figure S1E iii; G1∼3, G6, G7) but none in mammary glands of nulliparous WAP-*cre;Apc^CKO^* females (Figure S1E iii; G4, G5, G8, G9), confirming the specificity of the WAP promoter. A single prominent *Apc^Δ580^* band was detected by 3-allele screening PCR in four out of five WAP-*cre*-induced *Apc^CKO^* tumors irrespective of parity (Figure S1E ii; T5, T6, T7, T9), demonstrating the complete conversion of the conditional allele to the deleted allele. One WAP-*cre;Apc^CKO/+^* tumor from a multiparous female was heterozygous for *Apc^Δ580^* mutation with retention of the wild-type allele (Figure S1E ii; T3) while the other from a nulliparous female showed a reduced presence of the wild-type allele, suggesting an allelic loss (Figure S1E ii; T5). Those tumors that sporadically developed in *cre*-negative control mice were negative for the *Apc^Δ580^* allele (Figure S1E iii; T), implicating that their development was independent from Cre-induced *Apc* mutation. These genotyping results were further supported by RT-PCR of corresponding tumor RNA (data not shown).

To determine whether the inactivation of the remaining functional *Apc* allele was achieved by intragenic truncation mutations, we analyzed the tumor DNA by *in vitro* transcription and translation (IVTT) assay. In view of prior mutational analyses in humans and mice, the region of *Apc* considered most likely to contain mutations is the first 3 kb of exon 15 [19],[20]. All mammary tumors that showed retention of the wild-type *Apc* allele were analyzed by IVTT, and truncated Apc products were detected in 19 of 20 (95%) *K14-Cre*; *Apc^CKO/+^* and all 3 (100%) *Apc^Δ580/+^* mammary tumors, as well as in a single WAP-*Cre*; *Apc^CKO/+^* mammary tumor that did not show loss of the wild-type allele (Table 2). One sporadic *Apc^CKO/+^* mammary tumor contained two distinct *Apc* mutations. The relevant PCR products were subsequently cloned and sequenced. All 25 mutant sequences identified are shown in Table 2.

**Table 2:** Sequence of *Apc* mutations in various *Apc* heterozygous tumors.

Codon	Mutation	Consequence	Wild-type Sequence a	*K14-Cre*; *Apc^CKO/+^*	*WAP-Cre*; *Apc^CKO/+^*	*Apc^CKO/+^*	*Apc^Δ580/+^*
924–928	14 bpΔ	frameshift	AGA AGC --- --A CAC TCA	-	-	1^b^	-
1466	+C	frameshift	GAG AGT GGG C**C**T AAG CAG	1	-	-	-
1521	ΔAT	frameshift	GAT GTA GA**A T**TA AGA ATC	-	-	-	1
1528	C→T	Gln→Stop	CCT CCA GTT **C**AG GAA AAC	1	-	1^b^	-
1529	G→T	Glu→Stop	GTT CAG **G**AA AAC GAC AAT	1	-	-	-
1530	+A	frameshift	GTT CAG GAA A**A**C GAC AAT	2	-	-	-
1530	ΔAC	frameshift	GTT CAG GAA A**AC** GAC AAT	1	-	-	-
1534-1610	230 bpΔ	frameshift	AAT GGG A------ CCT GTG	1	-	-	-
1542	G→T	Glu→Stop	CAG CCT **G**AG GAA TCA AAT	1	-	-	-
1546	G→T	Glu→Stop	TCA AAT **G**AA AAC CAG GAT	-	-	-	1
1547	AC→T	frameshift	TCA AAT GAA A**AC** CAG GAT	1	-	-	-
1547	+A	frameshift	TCA AAT GAA A**A**C CAG GAT	1	-	-	-
1551	ΔA	frameshift	CAG GAT AAA G**A**G GTA GAA	1	-	-	-
1553	G→T	Glu→Stop	AAA GAG GTA **G**AA AAG CCT	1	-	-	-
1554	+A	frameshift	GAG GTA GAA A**A**G CCT GAC	1	-	-	-
1560-1565	14 bpΔ	frameshift	GAA AAA GA- --- -CT GAT	1	-	-	-
1561	A→T	Leu→Stop	TCT GAA **A**AA GAC TTA TTA	-	1	-	-
1567	ΔC	frameshift	TCT GAT GA**C** GAT GAT ATT	1	-	-	-
1567	ΔAC	frameshift	TCT GAT G**AC** GAT GAT ATT	1	-	-	-
1568	+T	frameshift	TCT GAT GAC+GAT GAT ATT	1	-	-	-
1568	ΔG	frameshift	TCT GAT **G**AC GAT GAT ATT	2	-	-	-
1570	ΔT	frameshift	GAT GAT AT**T** GAA ATA TTA	-	-	-	1
							
				19	1	2	3

a The wildtype sequence surrounding each mutation is shown and the site of mutation is shown in bold.

b Mutations found in the same mammary tumor.

When histologically distinct portions of a tumor were grossly identifiable as in the tumor in Figure 1C, they were collected separately and were analyzed by IVTT. It was found that they shared the same somatic truncation mutation, further supporting that these histologically distinct tumors derived from a clonal expansion of the same progenitor cell that have acquired an *Apc* truncation mutation (Figure 3B, T4&T5).

Most of mutations identified in mammary tumors were unique, and were previously not detected in intestinal tumors from *Apc^1638N/+^* heterozygous mice, with or without mismatch repair deficiency [19],[20]. All but four *Apc* truncation mutations detected in *K14-Cre*; *Apc^CKO/+^* mammary tumors were frameshift mutations (78.9%) of which two were intragenic deletions of over 10 bp. Most notably, despite the variety of mutant sequences, most of the mutations found were clustered further downstream, beyond codon 1500, than the mutation cluster region of *Apc* mutations (codons 850–1470) frequently found in mouse gastrointestinal tumors [19],[20] (Figure 3C). It is of interest to note that a sporadic *Apc^CKO/+^* mammary tumor contained two distinct *Apc* mutations that were located in very different regions of the *Apc* gene. One mutation would result in a truncated product that lacks all the β-catenin binding domains analogous to *Apc^Δ580^* mutation, while the other was located in the same region where mutations in *K14-Cre*; *Apc^CKO/+^* mammary tumors were found. These results indicate that not only the inactivation of the remaining wild-type allele of *Apc* is a pre-requisite in these tumors but there is also a selection for particular types of *Apc* somatic truncation mutations that are likely to result in some retention of down-regulating β-catenin signaling.

### Activating Ras Mutations in *K14-cre*; *Apc^CKO/+^* Mammary Tumors

*Wnt1*-induced mammary tumors frequently contain activating *H-Ras* mutations [21] and mutations in *K-Ras* or *N-Ras* are frequently found in *c-Myc*-induced tumors [22]. To determine whether secondary somatic mutations in *Ras* are involved in *Apc* mutation-induced mammary tumorigenesis, cDNA copies of tumor *Ras* mRNAs were analyzed by direct sequencing. In our sequence-based studies of 17 *K14-cre*; *Apc^CKO/+^*, five WAP-*cre*; *Apc^CKO^* (*Apc^CKO/CKO^* and *Apc^CKO/+^* combined), and three *Apc^Δ580/+^* mammary tumors, activating mutations were found at codons 12 and 61 of either *H-Ras* or *K-Ras* only in a subset (7 of 17) of *K14-cre*; *Apc^CKO/+^* tumors but none from other models. There were four mutations in *K-Ras* and two in *H-Ras* and these mutations were mutually exclusive (Figure 3D, Table 3). It is of interest that, although the incidence is low, both *H*- and *K-Ras* activation mutations were found in *K14-cre*; *Apc^CKO/+^* mammary tumors, since *K-Ras* mutations were frequently found in *Myc*-induced mammary tumors but not *H-Ras* and vice versa in *MMTV-Wnt1* tumors. This further supports the molecular diversity as well as the histological heterogeneity of *K14-cre*; *Apc^CKO/+^* mammary tumors. *H-Ras* mutations were found in two of four tumors that were predominantly of acinar histology, whereas *K-Ras* mutations were found in tumors that either predominantly composed of undifferentiated or mixed with undifferentiated histology (4 of 8). Sequence-based analysis of exons 5 to 8 of the *Tp53* gene was also carried out but *Tp53* missense mutations were not detected.

**Table 3:** Histology and *Ras* mutations in mammary tumors.

Genotype	Mammary Tumors	N	*H-Ras*	*K-Ras*
*K14-cre; Apc^CKO/+^*	Acinar/Glandular	6	2 (Q61L)	0
	Basosquamous/Pilar	3	0	0
	Undifferentiated	2	0	1 (Q61R)
	Mixed	6	0	3 (G12D, G12V)
	Total	17	2	4
*Apc^Δ580/+^*	Basosquamous/Pilar	3	0	0
*WAP-cre; Apc^CKO^*	Acinar/Glandular	4	0	0
	Mixed	1	0	0
	Total	5	0	0

### Comparative Analysis of Expression Profiles of *K14-cre*; *Apc^CKO/+^* Mammary Tumors to Other Models

**Figure 4:**
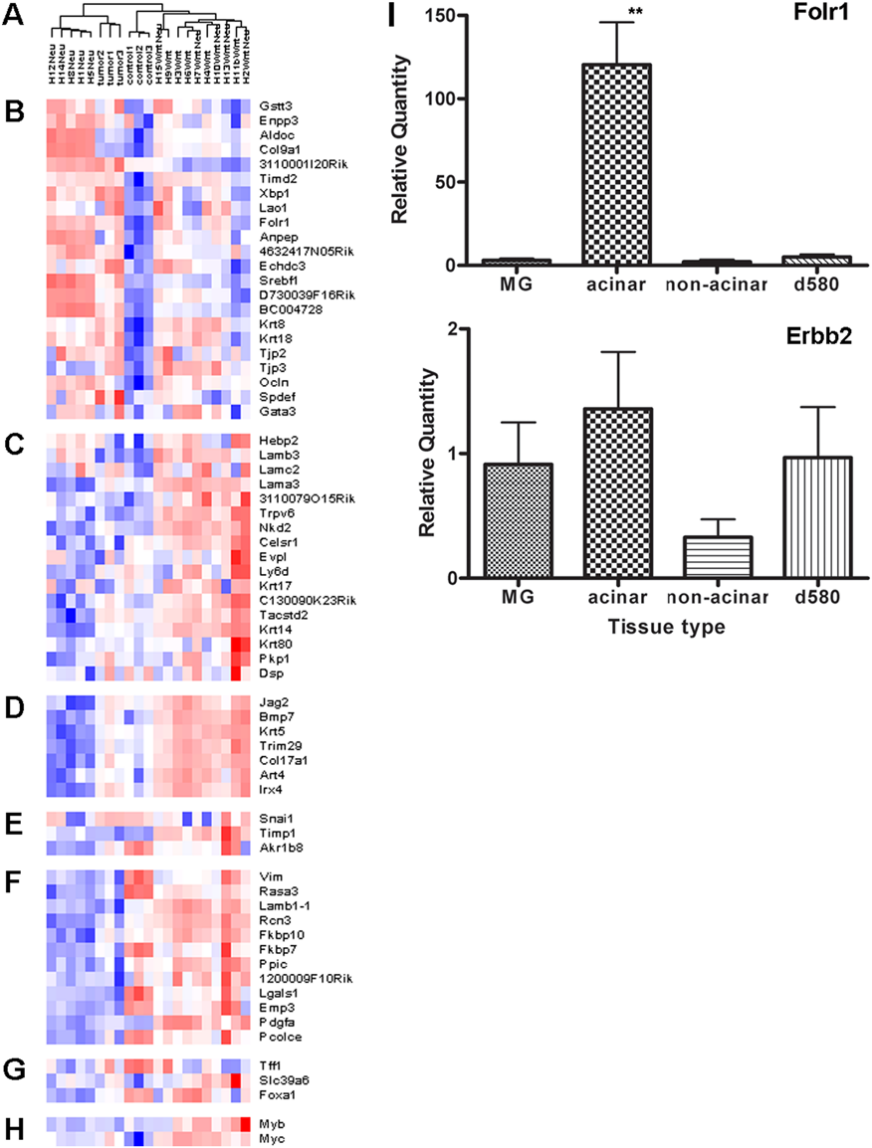
Comparison of expression profiles of *K14-cre*; *Apc^CKO/+^* mammary tumors to *MMTV-Wnt1* and *MMTV-Neu* tumors. (A) Dendrogram of *K14-cre*; *Apc^CKO/+^*, *MMTV-Wnt1*, *MMTV-Neu* and *MMTV-Wnt1/Neu* tumors [26], based on hierarchical clustering analyis of Affymetrix microarray data. *K14-cre*; *Apc^CKO/+^* acinar mammary tumors (tumor 1, 2, 3) showing more correlation to *MMTV-Neu*. (B–G) Mouse models intrinsic gene set cluster analysis, as depicted in Herzchkowitz et al [25]. Red and blue indicate expression levels respectively above and below the median. The magnitude of deviation from the median is represented by the color saturation. (B) Luminal epithelial gene expression pattern. (C) Genes encoding components of basal lamina. (D) A second basal epithelial cluster genes. (E) Genes implicated in epithelial to mesenchymal transition. (F) A second mesenchymal cluster that is expressed in normal. (G) Estrogen-regulated genes. (H) Potentially estrogen-regulated genes. (I) Real-time RT-PCR analysis of *Folr1* and *Erbb2* transcript levels in mammary tumors of the *K14-cre*; *Apc^CKO/+^* and *Apc^Δ580/+^* mice. Bars represent the mean values±S.E.M. for 7 control mammary glands (MG), 12 acinar and 6 non-acinar (undifferentiated or basosquamous) *K14-cre*; *Apc^CKO/+^* and 4 *Apc^Δ580/+^* (d580) mammary tumors normalized to the *Hprt1* transcript levels. The difference in the *Folr1* transcript levels was statistically significant between the control only in the acinar-type *K14-cre*; *Apc^CKO/+^* mammary tumors (p<0.006), whereas no difference was detected for the *Erbb2* transcript levels between the tumors and the control.

There are many mouse models of breast cancer that are different in histopathology and possibly in cell of origin [23],[24] and these models also have distinct gene expression profiles [25],[26]. To determine what types of mammary tumor models *K14-cre*; *Apc^CKO/+^* mice represent, gene expression profiles of three acinar-type mammary tumors, the most frequently found histological pattern in *K14-cre*; *Apc^CKO/+^* in the mixed background, and three *cre*-negative normal mammary gland samples were determined using Affymetrix GeneChip M430 2.0 arrays. All 3 tumors were heterozygous for *Apc^Δ580^* mutation and the remaining allele contained a truncation mutation in *Apc*. Both Gene Set Enrichment analysis (GSEA) and Ingenuity Pathway analysis (IPA) results indicated that mouse tumor profiles have gene sets characteristics of cell cycle, cellular movement and cancer related genes (Dataset S3, S4, S5, and S6). To get additional insights into *K14-cre*; *Apc^CKO/+^* tumors, gene expression data from our model was compared to data set of multiple mouse mammary carcinoma models previously published [25]. Based on mouse model intrinsic gene set cluster analysis [25], a dendrogram and a heatmap were generated using dChip (http://biosun1.harvard.edu/complab/dchip/) (Figure 4A–H). The gene expression pattern in dendrogram showed more correlations with luminal-type mammary tumors, which include *MMTV-Neu*, *MMTV-PyMT* and WAP-*Myc* [25]. *K14-cre*; *Apc^CKO/+^* tumors also expressed genes that are strongly expressed in human luminal tumors, such as *XBP1* and luminal cell marker K8 and K18, but were low in basal tumor-defining genes. These tumors also showed high expression of *Folate receptor 1* (*Folr1*), which is commonly up-regulated in luminal tumor mouse models [25]. As with most mouse mammary tumors, our model was also negative for *ER* and many estrogen-regulated genes. In agreement with these data, *K14-cre*; *Apc^CKO/+^* acinar-type mammary tumors expressed K8 while K14 or α-SMA staining was restricted to a myoepithelial pattern (Figure 2B–D).

To confirm the initial comparative results, we further compared the gene expression of our *K14-cre*; *Apc^CKO/+^* model to those previously published for *MMTV-Neu*, *MMTV-Wnt1* and *MMTV-Wnt1/Neu* bitrangenic mice [26]. A clustering diagram was obtained that clustered tumors from *MMTV-Neu*, *MMTV-Wnt1* and *MMTV-Wnt1/Neu* bitrangenic samples the same way as indicated previously [26], with *K14-cre*; *Apc^CKO/+^* tumors clustering in a separate cluster when hierarchical clustering analysis was done by samples using rank correlation as distance measure for 19,581 probes. However if differentially expressed probes from *K14-cre*; *Apc^CKO/+^* tumors versus controls were used for clustering, *K14-cre*; *Apc^CKO/+^* tumors clustered next to *MMTV-Neu* with no effect on clustering between *MMTV-Neu*, *MMTV-Wnt1* and *MMTV-Wnt1/Neu* samples. The same overall clustering was obtained using mouse model intrinsic gene set [25] (Figure 4A–H). These results support the view that acinar-type mammary tumors from *K14-cre*; *Apc^CKO/+^* model are luminal type that correlate more with *MMTV-Neu* than *MMTV-Wnt1* model.

The elevated expression of *Folr1* in mammary tumors detected by microarray analysis was confirmed by quantitative real-time RT-PCR. Since both the IPA and clustering analyses suggested a potential involvement of *Neu/Erbb2* (NM_001003817) in *K14-cre*; *Apc^CKO/+^* mammary tumorigenesis (Figure S3A), we also included *Erbb2* in our analysis. Over 30-fold increase in *Folr1* (NM_008034) expression compared to the control was detected in acinar-type mammary tumors (108.7±23.3 vs 3.1±1.1, p = 0.0051) confirming the microarray results, but non-acinar type, including those tumors composed primarily of basosquamous, pilar, and undifferentiated structures, and *Apc^Δ580/+^* mammary tumors showed no such differences (Figure 4I). There were no significant differences between tumors and the control for *Erbb2* expression.

## Discussion

To delineate the role of *Apc* mutations in mammary gland, we used Cre-loxP technology to target inactivation of *Apc* gene in two different mammary epithelial cells, using *K14-cre* and WAP-*cre* transgenic mice. The Cre expression in our *K14-cre* transgenic mice is driven by the basal *K14* promoter, which is active in progenitor cells that can give rise to both mammary luminal and myoepithelial lineages [27],[28], whereas that of WAP-cre transgenic mice is specific to lactating luminal epithelial cells [17]. The availability of *Apc* mutant mice under two different mammary promoters and their mammary tumors allowed us to study how Apc loss contributes to mammary tumorigenesis. In this study, we present several lines of evidence that target cells for *Apc* mutation-induced mammary tumorigenesis are progenitor/stem cells and that they require specific truncation mutations that partially retain β-catenin down-regulating function. First, *K14-cre* induced *Apc^Δ580^* heterozygosity, but not WAP-cre induced *Apc^Δ580^* heterozygosity or homozygosity, predisposes to mammary tumorigenesis. Second, *K14-cre* mediated mammary adenocarcinoma showed mixed lineage differentiation, in line with stem or progenitor cell origin, in contrast to WAP-*cre* mediated tumors that comprised essentially of luminal and abnormal α-SMA positive cells, lacking other basal markers. This is further supported by the fact that two grossly and histologically distinct regions of a tumor share the same somatic *Apc* truncation mutation, suggesting their origin from a common progenitor. Finally, the remaining wild-type allele of *Apc* is inactivated not by allelic loss, which is the common mechanism in intestinal tumorigenesis in *Apc* heterozygous mice, but preferentially by somatic truncation mutations specifically in a well defined region of the gene. This mutation cluster region was different to the one reported for intestinal tumors, implicating that the dosage-specific activation of downstream Wnt/β-catenin signaling pathway is necessary for mammary tumorigenesis.

We have previously shown that *K14-cre;Apc^CKO/CKO^* mice have aberrant development and squamous metaplasia in many epithelial-derived tissues and die perinatally [14], not allowing the analysis of Apc loss in postnatal mammary gland. Analogous to *K14*-driven *Apc^Δ580^* homozygosity, a complete inactivation of the *Apc* gene in WAP-expressing mammary luminal epithelial cells primarily led to the development of severe squamous metaplasia but rarely neoplasia. These observations suggest that constitutive activation of Wnt/β-catenin signaling pathway by Cre-mediated Apc deficiency, resulting in homozygous *Apc* (*Apc^Δ580/Δ580^*) mutations, invariably induce terminal squamous transdifferentiation of the mammary epithelium irrespective of cell origin of mutated cell, but do not develop tumors. This is in agreement with other Cre-mediated Wnt/β-catenin activation models in which induction of squamous metaplasia but not neoplasia was primarily observed [12],[13],[29].

Although homozygous *Apc* (*Apc^Δ580/Δ580^*) mutations induced either by *K14* or WAP-promoters invariably results in squamous metaplasia, Cre-mediated *Apc* heterozygosity (*Apc^Δ580/+^*) in *K14-cre*; *Apc^CKO/+^* mice developed mammary tumors with high penetrance. A similar tendency was also observed in *K14-cre*; *Apc^CKO/+^* mice backcrossed to C57BL/6, suggesting that the initiation of *Apc*-mediated mammary tumorigenesis is not affected by the genetic background. The majority of mammary tumors developed in *K14-cre*; *Apc^CKO/+^* mice had *Apc^Δ580/+^* genotype and have somatically acquired truncation mutation in the remaining wild-type allele, unlike intestinal tumors in *Apc^Δ580/+^* mice and other germline *Apc* heterozygotes in which the preferential mechanism of the wild-type *Apc* is allelic loss [14], [30]–[32]. Most intriguingly, these truncation mutations were clustered around codon 1530 of Apc (Figure 3C, Table 2), which is further downstream than the mutation cluster region typically observed in both human and mouse intestinal tumors [19],[20],[33]. Regulation of intracellular β-catenin levels is thought to represent one of the most important functions of the Apc tumor suppressor protein. Three different motifs in the central region of Apc are responsible for this activity. The three 15-amino acid (aa) repeats that bind β-catenin, the seven 20-aa repeats that both bind and down-regulate β-catenin [34],[35], and the three SAMP motifs that bind conductin/axin [36],[37]. The mutations found in mammary tumors would result in truncated Apc polypeptides retaining up to three of the seven 20-aa repeats but lack all SAMP motifs. It has been shown that loss of these functional motifs, especially those that lead to the elimination of at least five of the seven 20-aa repeats greatly reduced the β-catenin down-regulation activity of Apc [38]. However, Smits et al [39] showed that haploinsufficiency for the truncated Apc polypeptide that retains up to the third 20-aa repeat (*Apc^1638N/1572T^*) still retained some β-catenin down-regulation activity, resulting in a 5-fold increase in the transcriptional activity compared to a 30-fold increase in *Apc* mutation homozygosity (*Apc^1638N/1638N^*).

Since dosage of β-catenin is critical in determining epithelial cell fate in many organs and varying the level of β-catenin signaling during a cell fate program have been shown to switch the epithelial cell fate [40], it is possible that the homozygosity of *Apc* mutation (*Apc^Δ580/Δ580^*) results in too much β-catenin transcriptional activity, that may push cells into the signaling events that leads to squamous transdifferentiation rather than to hyperplasia of mammary epithelial cells and eventually to neoplasia. The *Apc^Δ580^* heterozygosity with somatic mutation of the remaining wild-type *Apc* allele that retains some β-catenin down-regulating domains may lead to the optimal dosage of β-catenin necessary for mammary tumorigenesis. Indeed, *Apc^Δ580/Δ580^* homozygosity induced by *K14* promoter resulted in severe squmaous metaplasia and ectopic hair follicle morphogenesis in many organs including skin and thymus [14] and the current data using WAP-*cre* also resulted in squamous metaplasia in mammary glands rather than tumor development. Since mutations found in mouse intestinal tumors result in either allelic loss or a somatic truncation mutation upstream of the third 20-aa repeats [14], [19], [20], [30]–[32], deletion of all the β-catenin binding domains seems to confer the main selective advantage in mouse intestinal tumorigenesis. Thus, our results and those of others indicate that there is an Apc-regulated level of β-catenin signaling optimal for tumor formation that differs tissue-specifically. The selection for an optimal β-catenin signaling level for tumor formation is also supported by the spectrum of somatic mutations observed in colorectal adenomas from Familial Adenomatous Polyposis (FAP) patients with different germline mutations in *APC* [41]. Our data also partly explain why breast cancers do not develop as frequently as colorectal tumors in FAP patients. It is likely that unless somatic truncation mutation optimal for breast oncogenesis is acquired in the *APC* gene, mammary epithelial cells are not initiated towards tumorigenesis but instead become metaplastic. Such selection would reduce the incidence of tumorigenesis and require much longer latency, as demonstrated by *Apc^Δ580/+^* and *K14-cre*; *Apc^CKO/+^* mouse models.

Our histopathological and molecular analyses showed that the majority of *K14-cre;Apc^CKO/+^* mammary tumors are adenocarcinomas with multiple foci of squamous metaplasia. These tumors are highly proliferative, *ER*-negative carcinomas, showing strong positivity for Tcf/β-catenin target genes, Myc and cyclin D1, with expression of both luminal and basal epithelial markers. They have many histological features common to Wnt pathway tumors previously described [18], but at the same time have expression profiles that correlate more to luminal tumor models. The latter observation could be explained partly by the predominance of acinar-type histology, which is a luminal type histology, found in the majority of *K14-cre;Apc^CKO/+^* tumors from the mixed background and was the histological type selected for the expression analysis in the current study. The selection of this particular histological type may have biased the expression profiles towards luminal expression pattern. Interestingly, the frequency of the acinar-type histology diminished and basosquamous/pilar structures predominated in the *K14-cre;Apc^CKO/+^*-B6 tumors. Since either histological type of mammary tumors had the same mechanism of Apc inactivation irrespective of the genetic backgrounds, it suggests that the mode of initiation is the same but the progression to certain histological types is greatly influenced by modifier genes associated with the genetic backgrounds.

Comparison of our gene expression profiling data to published mouse and human breast cancers suggests that acinar-type tumors from *K14-cre;Apc^CKO/+^* mice are more similar to luminal type mouse models [25]. The similarities between mouse and human luminal tumors are limited by the fact that most human luminal epithelial cluster contains the *ER* and many estrogen-regulated genes, but many mouse mammary tumors, including *K14-cre;Apc^CKO/+^* tumors, are ER-negative. However, the expression profiles of *K14-cre;Apc^CKO/+^* acinar tumors also included a human luminal tumor-defining gene, *XBP1* [42],[43] and stained positive for K8. Our tumor set also showed elevated expression of *Folr1*, which is a gene included in luminal epithelial gene expression cluster that is highly expressed in *MMTV-PyMT*, *MMTV-Neu*,and *WAP-myc* tumors [25]. It was of interest that *Folr1* expression level varied within *K14-cre;Apc^CKO/+^* mammary tumors depending on histology, and the ones that had elevated expression were those predominantly composed of acinar histology. The tumors from *Apc^Δ580^* mice, which were of pilar and basosquamous histology, had very low *Folr1* expression level. Although *Folr1* is not associated with human luminal tumors, its overexpression and poor prognosis have been implicated in human breast cancers [44],[45].

To determine whether other oncogenic pathways are involved in *Apc* mutation-induced mammary tumorigenesis, we examined the status of *Ras* oncogenes and *Tp53* in these tumors. It has been previously shown that *c-Myc* induced mammary tumors in mice frequently harbor spontaneous activating mutations in *K-Ras* [22] while over 50% of *MMTV-Wnt1* tumors contain oncogenic mutations in *H-Ras* [21]. Jang et al also suggested that *K-Ras* activation strongly synergizes with both *c-Myc* and *Wnt1* in mammary tumorigenesis and promotes the progression of tumors to oncogene independence, while *H-Ras* mutant *Wnt1*-induced tumors remain oncogene dependent [46]. It is of interest that although their presence was mutually exclusive to each other, both *H-Ras* and *K-Ras* oncogenic mutations were found in a subset *K14-cre;Apc^CKO/+^* tumors. We could not find a strong association between histology, incidence of lung metastasis and *Ras* mutations, but those tumors that contained *K-Ras* mutations frequently had heterogeneous histology containing aggressive looking undifferentiated regions that have lost both lineage markers while those with *H-Ras* mutations were predominantly of acinar-type.

In conclusion, our study demonstrates that activation of Wnt/β-catenin signaling via inactivation of *Apc* leads to mammary tumorignesis when the inactivation takes place in mammary epithelial progenitor cells rather than more differentiated secretory luminal cells; and when somatic truncation mutation is acquired in a particular region of the *Apc* gene, which may be necessary to achieve a certain level of β-catenin signaling activation required for mammary tumorigenesis. These initiated tumor cells develop into heterogeneous tumor containing different histological types, each with different expression pattern of lineage markers, some acquiring more oncogenic mutations, such as in either *H-Ras* or *K-ras* genes. Our data indicate that only a specific subset of somatic mutations at the *Apc* gene will successfully lead to tumor formation in the mammary epithelium and there is a selection for *Apc* mutations that retain some down-regulating activity of β-catenin signaling. We propose that this selection is aimed at a specific level of β-catenin signaling optimal for mammary tumorigenesis, rather than at its constitutive activation achieved by deletion of all the β-catenin down-regulating domains in *Apc*, which invariably results in squamous metaplasia.

## Materials and Methods

### Mammary-Specific Inactivation of *Apc*

The *Apc* conditional (*Apc^CKO^*) and germline (*Apc^Δ580^*) knockout mice, WAP-*cre* and *K14-cre* transgenic mice have previously been described [14],[16],[17]. *Apc^Δ580/+^* mice have already been backcrossed to C57BL/6 background. The rest of the mice analyzed in this study were generated as follows: *Apc^CKO^* heterozygote mice of the F1 generation (C57BL/6×129/S background) were first crossed with either WAP-*cre* mice (C57BL/6 background) or *K14-cre* transgenic mice (FVB background). *Cre*-positive *Apc^CKO/+^* male mice thus generated were then crossed with *Apc^CKO/CKO^* females to generate homozygous and heterozygous *Apc^CKO^* offspring either with or without respective *cre* transgene. The mice were intercrossed thereafter for maintenance. Subsequently, *K14-cre*; *Apc^CKO/+^* female mice backcrossed for 4 generations to achieve >95% C57BL/6J background were also included in the analysis so that the results between two different promoters will be comparable. Females with genotypes WAP-*cre*; *Apc^CKO/+^* and WAP-*cre*; *Apc^CKO/CKO^* were mated and undergone pregnancies 4 times to facilitate WAP-*cre*-mediated deletion of exon 14 of *Apc* gene. Mice with genotype *K14-cre*; *Apc^CKO/CKO^* are perinatally lethal [14] and phenotypically normal *K14-cre*; *Apc^CKO/+^* littermates were used for analysis. The mice were sacrificed when either they were moribund or their tumors reached at least 2 cm in diameter, following Institutional Animal Care and Use Committee guidelines.

### Genotyping of Mice

Mouse tails tips obtained at ∼10 days of age were lysed overnight in DirectPCR Lysis Reagent (Viagen Biotech) containing 0.1 mg/ml Proteinase K (Qiagen). The crude lysates were incubated at 85°C for 45 minutes and 0.5 µl of lysate was directly used per 25 µl PCR reaction. Detection of various *Apc* alleles and *cre* transgene was carried out as previously described [14].

### Histological Analysis

Mice were sacrificed by CO_2_ inhalation when they developed gross tumors or were moribund. The location and size of tumors were routinely recorded and pictures were taken from each mouse. Tumors were cut in portions and a portion was either fixed in 10% netural buffered formalin (NBF) or in 4% paraformaldehyde. The other portion was either snapped frozen in liquid nitrogen or immersed in RNAlater solution (ambion) overnight and stored at −80°C until molecular analyses. Mammary glands were also collected routinely from each mouse: 4^th^ mammary gland was fixed flat on a piece of paper towel in 10% NBF, 9^th^ mammary gland for whole mount and either 8^th^ and 3^rd^ tumor-free mammary gland was collected for molecular analyses. The mice were then dissected for gross examination. A portion of liver and lungs were similarly collected and fixed. Then the whole body was fixed in Bouin's solution. The fixed tissue samples were then submitted to Rodent Histopathological Core, processed and embedded in paraffin. Tissue sections were cut and stained with H&E for histopathological examinations.

### Immunohistochemistry

Five-µm sections were cut from the paraffin-embedded tissues and immunohisochemistry was performed essentially as previously described [14]. Briefly, sections were deparaffinized, rehydrated and boiled in either Citrate buffer (10 mM, pH 6) or Tris buffer (10 mM Tris, 1 mM EDTA, pH 9) for antigen retrieval. Slides were than treated with 3% peroxidase in PBS, followed by blocking in normal horse serum. Primary antibodies against Ki67 (1∶200, Vector Laboratories), β-catenin (1∶200, BD Transduction Lab), cyclin D1 (1∶100, Lab Vision), c-myc (1∶200, Upstate), esterogen receptor ERα and progesterone receptor (1∶2000, 1∶500, respectively, Santa Cruz) and cellular markers such as cytokeratins K1, K6, K14 (1∶1000, 1∶500, 1∶2000, respectively, Covance), K8 (1∶100, TROMA-I, DSHB), p63 (1∶200, Chemicon International) and α-smooth muscle actin (α-SMA, 1∶800, Sigma) were applied followed by an incubation with biotin-conjugated appropriate secondary antibody. Mouse-on-Mouse kit (Vector Laboratories) was used with the mouse primary antibodies. The Vectastain Elite ABC kit and DAB (Vector Laboratories) were used for detection, following manufacturer's instructions.

### Mammary Gland Wholemount

This procedure was carried out as described on the mammary gland website: http://mammary.nih.gov.

### DNA and RNA Extraction from Tumors

Both genomic DNA and RNA from tumors and various tissue samples collected at the time of autopsy were extracted as described previously [14]. Briefly, genomic DNA was extracted using DNeasy mini kit (QIAGEN). RNA was extracted by homogenizing tumors and tissues in 3 ml Trizol reagent (Invitrogen). After phase separation, an equal volume of 70% ethanol was added to the aqueous phase and purified through PureLink Micro-to-Midi Total RNA Purification System (Invitrogen), following manufacturer's instruction. Concentrations of nucleic acids were determined by Nanodrop (Ribogreen, Molecular Probes, Eugene, Oregon, United States).

### Analysis of *Apc* Status in Tumors

Tissue-specific recombination of the conditional alleles in tumors and various tissue samples was examined by analyzing both their extracted DNA and RNA as described previously [14]. Briefly, genomic DNA samples were examined by a semi-quantitative 3-primer genotyping PCR that will give 3 distinct sized products from the wild-type (320 bp), conditional (430 bp) and deleted (500 bp) alleles of *Apc*. In addition, two separate 2-primer PCRs were performed; one to check for the wild-type and *Apc^CKO^* alleles and the other to detect the presence of the *Apc^Δ580^* allele alone. The expression of full-length and truncated *Apc* alleles were examined by performing RT-PCR on RNA using SuperScript One-Step RT-PCR with Platinum Taq (Invitrogen), following manufacturer's protocol.

Upon confirmation of the retention of the wild-type *Apc* allele, codons 677–1674 of the mouse *Apc* gene were analyzed for truncation mutations by PCR and *in vitro* transcription and translation (IVTT) assay as described previously [20],[47] but with some modifications. All DNA amplifications were performed using Pfu Ultra II fusion HS DNA polymerase (Stratagene) according to manufacturer's instruction. The wild-type allele-specific amplification of *Apc* was performed by nested-PCR to eliminate co-amplification of deleted *Apc^Δ580^* allele using the forward primer 5′-CATTCTCCCCTACTTAGATGG and a reverse primer 5′-GTTGTCATCCAGGTCTGGTG in the first PCR reaction. Two overlapping segments of the *Apc* gene covering codons 677–1234 and 1100–1674 were subsequently amplified from aliquots of the first reactions using two pairs of PCR primers specific for IVTT. Cycling conditions for the first stage PCR were one cycle of 94°C for 2 min, followed by 20 cycles of 94°C for 20 s, 58°C for 20 s and 72°C for 90 s, with one final extension cycle at 72°C for 3 min. Cycling conditions for the second stage PCR were as above except the cycle number was increased to 25. The PCR products were directly used for IVTT assay as described previously [20],[47]. In order to facilitate the detection of *Apc* truncation mutations from tumor samples that are frequently co-harvested with adjacent normal tissues, we developed a method based on expression of Apc-GFP fusion polypeptides in bacteria, in which colonies derived from PCR products with mutation appear GFP-negative. The pTrcHis B Prokaryotic Expression Vector (Invitrogen) was modified to contain GFP coding sequence (Acc# U87625) between NheI and SacI sites. The sequence between NcoI and NheI sites, coding poly-histidine region was replaced with a BamHI site, such that insertion of amplified *Apc* fragments between BamHI and NheI sites restores the reading frame of GFP. For characterization of tumor-specific mutations, the PCR products were digested with *Bam*HI and *Nhe*I, gel purified, and cloned into modified pTrc vector and transformed into bacterial cells using standard cloning procedures. Transformed bacterial cells were spread on LB plates containing final concentrations of 50 µg/ml ampicillin and 50 µM IPTG and incubated overnight at 37°C. The numbers of non-fluorescent and fluorescent colonies were counted under long-wave length UV light. When the percentage of non-fluorescent colonies over total was above the control level, individual GFP negative clones were screened by IVTT to identify mutations, and their DNA sequences were determined.

### *Tp53* and *Ras* Mutational Analysis

For p53 mutational analysis, exons 5 to 8 of *Tp53* were examined by performing RT-PCR on RNA using primers p53-F117 and p53-R313 and SuperScript One-Step RT-PCR with Platinum Taq (Invitrogen). The PCR products were then sequenced by nested primers, p53-F121 and p53-R308. For *K-Ras* and *H-Ras* codons 12, 13 or 61 mutation analysis, RNA was first reverse-transcribed and amplified using SuperScript One-Step RT-PCR system, followed by nested-PCR using Pfu Ultra II fusion HS DNA polymerase (Stratagene). The mutations were scored positive when approximately half of the resulting amplified DNA had the same mutation, and when the results were confirmed by either sequencing from both ends or by sequencing corresponding genomic amplified products. Sequences of primers used in the study are listed in Table S1.

### Gene Expression Profiling

Total RNA (5–10 µg) extracted from 3 acinar-type mammary tumors and 3 age-matched control mammary glands from *cre*-negative mice were hybridized and scanned to GeneChip M430 2.0 according to Affymetrix protocols (Affymetrix). Scanned microarray images were imported into GeneChip Operating Software (GCOS, Affymetrix) to generate signal values and absent/present calls for each probe-set using the MAS 5.0 statistical expression algorithm (.chp files). Each array was scaled to a target signal of 500 using all probe-sets and default analysis parameters. Prior to performing any other analysis Affymetrix detection calls were used to remove 12,844 probes which had ‘Absent’ call across all samples. Data set with 32,322 probes was used as starting point for any subsequent analysis. To identify genes differentially expressed between tumor and control samples two-sample t-test was used (Dataset S1). T-test was performed using ComparativeMarkerSelection module of Gene Pattern (http://www.broad.mit.edu/cancer/software/genepattern/) [48]. Based on two-sample t-test results, any probes with fold differences below 2, t-test values below 4.5 were removed and only the probes that have either consistent absent or present calls were used as input for Ingenuity Pathway Analysis (IPA) Software (http://www.ingenuity.com/, Dataset S2). Ingenuity core analysis generated over 60 networks with nearly 600 network nodes in total, many of them involved in cell cycle, cell, growth, cell death, DNA replication, and cancer (Dataset S3, S4, S5, and S6).

Gene Set Enrichment Analysis (GSEA) was performed using java GSEA http://www.broad.mit.edu/gsea/ as previously described [49]

### Comparison of Gene Expression Profiles with Other Mouse Models

To get additional insights in *K14-cre*; *Apc^CKO/+^* tumors, gene expression from our mouse model was compared to data set of multiple mouse mammary carcinoma models and human breast tumors previously published [25]. In particular, based on genes mainly in intrinsic gene set cluster analysis of mouse models a dendrogram was generated using dChip (http://biosun1.harvard.edu/complab/dchip/) (Figure 4A).

To further compare gene expression of *K14-cre*; *Apc^CKO/+^* model, gene expression probe level data (CEL files 430A 2.0) from Shixia Huang et al. for tumors from *MMTV-Wnt1*, *MMTV-Neu* and *MMTV-Wnt1/MMTV-Neu* bitransgenic mice were obtained [26]. MAS5.0 algorithm was used to estimate probe expression. Since 430 2.0 chips have additional probe sets compared to 430A 2.0 the subset corresponding to 430A 2.0 was used for MAS5.0 global method of scaling/normalization. Target Intensity value of 500 was used for all arrays. Prior to performing any other analysis, Affymetrix detection calls were used to remove 3,045 probes which had ‘Absent’ call across all samples. Data set with 19,581 probes was used as starting point for any subsequent analysis. Hierarchical clustering by samples was performed using data for all 19,581 probes as input and rank correlation as (Figure S3B). Subsequently, 1,335 differentially expressed probes (fold differences above 2, t-test values above 4.5) from *K14-cre*; *Apc^CKO/+^* tumors versus controls were used for clustering analysis (Figure S3C).

### Real-Time RT-PCR

The expression levels of *Folr1* and *Erbb2* in 17 *K14-cre*; *Apc^CKO/+^*, 5 WAP-*cre*; *Apc^CKO^* (*Apc^CKO/CKO^* and *Apc^CKO/+^* combined), 4 *Apc^Δ580/+^* mammary tumors were compared to those of 7 normal mammary glands by quantitative RT-PCR with *HPRT1* as an internal control. Most of *K14-cre*; *Apc^CKO/+^* mammary tumors in the mixed background consisted of acinar-type, but there were a few which had distinct histology. Therefore, tumors were roughly divided into 2 groups; acinar and non-acinar. The latter group of mammary tumors primarily composed of undifferentiated, basosquamous and pilar-type histology. We analyzed 14 acinar-type, and 8 non-acinar type. The mammary tumors developed in *Apc^Δ580/+^* mice were mostly of basosquamous-type. TaqMan Gene Expression Assays for respective genes were used on 7500 Fast Real-Time PCR System (Applied Biosystem) according to the manufacturer's protocol. Relative quantity was calculated using Sequence Detection Software version 1.4 (Applied Biosystem).

## Supporting Information

Figure S1 WAP-*cre* induced inactivation of *Apc*. (A) Whole-mount of a mammary gland showing development of severe squmaous metaplasia in multiparous WAP-*cre*; *Apc^CKO/CKO^* female mouse. (B) H&E staining of another gland from the same mouse showing multiple metaplstic lesions throughout the gland. (C) H&E staining of a mammary tumor from a multiparous WAP-*cre*; *Apc^CKO/CKO^* female mouse with squamous metaplasia (arrows) which have less defined structures than those in *K14-cre*; *Apc^CKO/+^* tumors. (D) K14 expression was only observed in these metaplstic lesions in WAP-*cre* induced tumors. Scale bars: 50 µm. (E) Genotyping for *Apc* in WAP-*cre* positive mammary glands and tumors, showing WAP-cre and parity-specific recombination of conditional alleles. Genotyping PCR for the (i) wild-type and *Apc^CKO/+^* alleles, (ii) wild-type, *Apc^CKO/+^* and *Apc^Δ580^* alleles, (iii) *Apc^Δ580^* allele alone. The presence of the *Apc^Δ580^* allele detected in mammary tumors from nulliparous WAP-cre positive females demonstrates that these tumors have derived from a clone of cells that have undergone Cre-mediated recombination.
(3.0 MB PPT)

Figure S2 Expression of Tcf/β-catenin-target genes in mammary tumors from *K14-cre*; *Apc^CKO/+^* female mice. A representative acinar-type mammary tumor stained with H&E for histology (A), luminal marker K8 (B), basal marker K14 (C) and β-catenin (D), showing strong positivity for Tcf/β-catenin-target genes cyclin D1 (E) and c-myc (F, G). Examples of cells with strong c-myc positivity are indicated by arrows (G). Scale bars: 50 µm for (A–F), 25 µm for (G).
(7.8 MB PPT)

Figure S3 Ingenuity Pathway and Hierarchical Clustering analyses of expression profiles of *K14-cre*; *Apc^CKO/+^* mammary tumors. (A) A representative IPA network. Ingenuity core analysis generated over 60 networks, many of them involved in cell cycle, cell, growth, cell death, DNA replication and cancer (see Dataset S3 for information for all networks). A representative network is shown, showing elevated expression of *Folr1*. Green for elevated expression in tumor and red for elevation in control. (B) Hierarchical clustering by samples for 19,581 probes. A clustering diagram of *K14-cre*; *Apc^CKO/+^* tumors (1107 E,F,H), controls (1107 A,C,D), tumors from *MMTV-Wnt1*, *MMTV-Neu* and *MMTV-Wnt1/MMTV-Neu* bitransgenic mice. Rank correlation was used as distance measure. (C) Hierarchical clustering by samples for 1,335 probes. 1,335 differentially expressed probes (fold differences above 2, t-test values above 4.5) from *K14-cre*; *Apc^CKO/+^* tumors versus controls were used.
(0.5 MB PPT)

Table S1 Primer Sequences for *Tp53* and *Ras* Mutational analysis.
(0.05 MB DOC)

Dataset S1 Differential gene expression between tumors and control samples. Information on differential gene expression between tumors and control samples for 32,322 probes.
(21 MB XLS)

Dataset S2 Input list of probes for Ingenuity Pathway Analysis. Information for 2,800 probes which were used as input for IPA. Out of data set with 32,322 probes that have been analyzed for differential expression by Gene Pattern, probes with fold differences below 2, t-test values below 4.5 were removed and only the probes that have either consistent absent (a) or present (p) calls, but not absent across all samples, i.e., ppp-ppp, aaa-ppp or ppp-aaa were used as input for Ingenuity Pathway Analysis Software. Overall 2,800 probes were used as input for IPA.
(2.7 MB XLS)

Dataset S3 Networks generated by Ingenuity Pathway Analysis. Information for all networks generated by Ingenuity Pathway Analysis.
(0.04 MB XLS)

Dataset S4 Molecules used in networks generated by Ingenuity Pathway Analysis. Information for all molecules used in networks generated by Ingenuity Pathway Analysis.
(0.7 MB XLS)

Dataset S5 Canonical Pathways. Information for Canonical Pathways identified by Ingenuity Pathway Analysis.
(0.05 MB XLS)

Dataset S6 Functions. Information on processes identified by Ingenuity Pathway Analysis.
(0.2 MB XLS)

## References

[1] Deugnier MA, Teuliere J, Faraldo MM, Thiery JP, Glukhova MA (2002). The importance of being a myoepithelial cell.. Breast Cancer Res.

[2] Turnbull C, Rahman N (2008). Genetic Predisposition to Breast Cancer: Past, Present, and Future.. Annual Review of Genomics and Human Genetics.

[3] Patocs A, Zhang L, Xu Y, Weber F, Caldes T (2007). Breast-Cancer Stromal Cells with TP53 Mutations and Nodal Metastases.. N Engl J Med.

[4] Mohinta S, Wu H, Chaurasia P, Watabe K (2007). Wnt pathway and breast cancer.. Front Biosci.

[5] Furuuchi K, Tada M, Yamada H, Kataoka A, Furuuchi N (2000). Somatic mutations of the APC gene in primary breast cancers.. Am J Pathol.

[6] Jin Z, Tamura G, Tsuchiya T, Sakata K, Kashiwaba M (2001). Adenomatous polyposis coli (APC) gene promoter hypermethylation in primary breast cancers.. Br J Cancer.

[7] Sarrio D, Moreno-Bueno G, Hardisson D, Sanchez-Estevez C, Guo M (2003). Epigenetic and genetic alterations of APC and CDH1 genes in lobular breast cancer: relationships with abnormal E-cadherin and catenin expression and microsatellite instability.. Int J Cancer.

[8] Moser AR, Mattes EM, Dove WF, Lindstrom MJ, Haag JD (1993). ApcMin, a mutation in the murine Apc gene, predisposes to mammary carcinomas and focal alveolar hyperplasias.. Proceedings of the National Academy of Sciences, USA.

[9] Michaelson JS, Leder P (2001). beta-catenin is a downstream effector of Wnt-mediated tumorigenesis in the mammary gland.. Oncogene.

[10] Imbert A, Eelkema R, Jordan S, Feiner H, Cowin P (2001). Delta N89 beta-catenin induces precocious development, differentiation, and neoplasia in mammary gland.. J Cell Biol.

[11] Teuliere J, Faraldo MM, Deugnier MA, Shtutman M, Ben-Ze'ev A (2005). Targeted activation of beta-catenin signaling in basal mammary epithelial cells affects mammary development and leads to hyperplasia.. Development.

[12] Miyoshi K, Shillingford JM, Le Provost F, Gounari F, Bronson R (2002). Activation of beta -catenin signaling in differentiated mammary secretory cells induces transdifferentiation into epidermis and squamous metaplasias.. Proc Natl Acad Sci U S A.

[13] Gallagher RC, Hay T, Meniel V, Naughton C, Anderson TJ (2002). Inactivation of Apc perturbs mammary development, but only directly results in acanthoma in the context of Tcf-1 deficiency.. Oncogene.

[14] Kuraguchi M, Wang XP, Bronson RT, Rothenberg R, Ohene-Baah NY (2006). Adenomatous Polyposis Coli (APC) Is Required for Normal Development of Skin and Thymus.. PLoS Genet.

[15] Owens DM, Watt FM (2003). Contribution of stem cells and differentiated cells to epidermal tumours.. Nat Rev Cancer.

[16] Jonkers J, Meuwissen R, van der Gulden H, Peterse H, van der Valk M (2001). Synergistic tumor suppressor activity of BRCA2 and p53 in a conditional mouse model for breast cancer.. Nat Genet.

[17] Wagner KU, Wall RJ, St-Onge L, Gruss P, Wynshaw-Boris A (1997). Cre-mediated gene deletion in the mammary gland.. Nucleic Acids Res.

[18] Rosner A, Miyoshi K, Landesman-Bollag E, Xu X, Seldin DC (2002). Pathway pathology: histological differences between ErbB/Ras and Wnt pathway transgenic mammary tumors.. Am J Pathol.

[19] Kuraguchi M, Edelmann W, Yang K, Lipkin M, Kucherlapati R (2000). Tumor-associated Apc mutations in Mlh1−/− Apc1638N mice reveal a mutational signature of Mlh1 deficiency.. Oncogene.

[20] Kuraguchi M, Yang K, Wong E, Avdievich E, Fan K (2001). The distinct spectra of tumor-associated Apc mutations in mismatch repair-deficient Apc1638N mice define the roles of MSH3 and MSH6 in DNA repair and intestinal tumorigenesis.. Cancer Res.

[21] Podsypanina K, Li Y, Varmus HE (2004). Evolution of somatic mutations in mammary tumors in transgenic mice is influenced by the inherited genotype.. BMC Med.

[22] D'Cruz CM, Gunther EJ, Boxer RB, Hartman JL, Sintasath L (2001). c-MYC induces mammary tumorigenesis by means of a preferred pathway involving spontaneous Kras2 mutations.. Nat Med.

[23] Cardiff RD, Moghanaki D, Jensen RA (2000). Genetically engineered mouse models of mammary intraepithelial neoplasia.. J Mammary Gland Biol Neoplasia.

[24] Hennighausen L (2000). Mouse models for breast cancer.. Breast Cancer Res.

[25] Herschkowitz JI, Simin K, Weigman VJ, Mikaelian I, Usary J (2007). Identification of conserved gene expression features between murine mammary carcinoma models and human breast tumors.. Genome Biol.

[26] Huang S, Podsypanina K, Chen Y, Cai W, Tsimelzon A (2006). Wnt-1 is dominant over neu in specifying mammary tumor expression profiles.. Technol Cancer Res Treat.

[27] Shackleton M, Vaillant F, Simpson KJ, Stingl J, Smyth GK (2006). Generation of a functional mammary gland from a single stem cell.. Nature.

[28] Stingl J, Eirew P, Ricketson I, Shackleton M, Vaillant F (2006). Purification and unique properties of mammary epithelial stem cells.. Nature.

[29] Meniel V, Hay T, Douglas-Jones A, Sansom OJ, Clarke AR (2005). Mutations in Apc and p53 synergize to promote mammary neoplasia.. Cancer Res.

[30] Luongo C, Moser AR, Gledhill S, Dove WF (1994). Loss of Apc+ in intestinal adenomas from Min mice.. Cancer Res.

[31] Oshima M, Oshima H, Kitagawa K, Kobayashi M, Itakura C (1995). Loss of Apc heterozygosity and abnormal tissue building in nascent intestinal polyps in mice carrying a truncated Apc gene.. Proc Natl Acad Sci U S A.

[32] Smits R, Kartheuser A, Jagmohan-Changur S, Leblanc V, Breukel C (1997). Loss of Apc and the entire chromosome 18 but absence of mutations at the Ras and Tp53 genes in intestinal tumors from Apc1638N, a mouse model for Apc-driven carcinogenesis.. Carcinogenesis.

[33] Miyoshi Y, Nagase H, Ando H, Horii A, Ichii S (1992). Somatic mutations of the APC gene in colorectal tumors: mutation cluster region in the APC gene.. Human Molecular Genetics.

[34] Rubinfeld B, Souza B, Albert I, Muller O, Chamberlain SH (1993). Association of the APC gene product with beta-catenin.. Science.

[35] Su LK, Vogelstein B, Kinzler KW (1993). Association of the APC tumor suppressor protein with catenins.. Science.

[36] Behrens J, Jerchow BA, Wurtele M, Grimm J, Asbrand C (1998). Functional interaction of an axin homolog, conductin, with beta- catenin, APC, and GSK3beta.. Science.

[37] Hart MJ, de los Santos R, Albert IN, Rubinfeld B, Polakis P (1998). Downregulation of beta-catenin by human Axin and its association with the APC tumor suppressor, beta-catenin and GSK3 beta.. Current Biology.

[38] Rubinfeld B, Albert I, Porfiri E, Munemitsu S, Polakis P (1997). Loss of beta-catenin regulation by the APC tumor suppressor protein correlates with loss of structure due to common somatic mutations of the gene.. Cancer Research.

[39] Smits R, Kielman MF, Breukel C, Zurcher C, Neufeld K (1999). Apc1638T: a mouse model delineating critical domains of the adenomatous polyposis coli protein involved in tumorigenesis and development.. Genes Dev.

[40] DasGupta R, Rhee H, Fuchs E (2002). A developmental conundrum: a stabilized form of beta-catenin lacking the transcriptional activation domain triggers features of hair cell fate in epidermal cells and epidermal cell fate in hair follicle cells.. J Cell Biol.

[41] Albuquerque C, Breukel C, van der Luijt R, Fidalgo P, Lage P (2002). The ‘just-right’ signaling model: APC somatic mutations are selected based on a specific level of activation of the beta-catenin signaling cascade.. Hum Mol Genet.

[42] Gruvberger S, Ringner M, Chen Y, Panavally S, Saal LH (2001). Estrogen receptor status in breast cancer is associated with remarkably distinct gene expression patterns.. Cancer Res.

[43] Sotiriou C, Neo SY, McShane LM, Korn EL, Long PM (2003). Breast cancer classification and prognosis based on gene expression profiles from a population-based study.. Proc Natl Acad Sci U S A.

[44] Hartmann LC, Keeney GL, Lingle WL, Christianson TJ, Varghese B (2007). Folate receptor overexpression is associated with poor outcome in breast cancer.. Int J Cancer.

[45] Kelemen LE (2006). The role of folate receptor alpha in cancer development, progression and treatment: cause, consequence or innocent bystander?. Int J Cancer.

[46] Jang JW, Boxer RB, Chodosh LA (2006). Isoform-specific ras activation and oncogene dependence during MYC- and Wnt-induced mammary tumorigenesis.. Mol Cell Biol.

[47] Kucherlapati M, Yang K, Kuraguchi M, Zhao J, Lia M (2002). Haploinsufficiency of Flap endonuclease (Fen1) leads to rapid tumor progression.. Proc Natl Acad Sci U S A.

[48] Reich M, Liefeld T, Gould J, Lerner J, Tamayo P (2006). GenePattern 2.0.. Nat Genet.

[49] Subramanian A, Tamayo P, Mootha VK, Mukherjee S, Ebert BL (2005). Gene set enrichment analysis: a knowledge-based approach for interpreting genome-wide expression profiles.. Proc Natl Acad Sci U S A.

[50] Chen PC, Kuraguchi M, Velasquez J, Wang Y, Yang K (2008). Novel roles for MLH3 deficiency and TLE6-like amplification in DNA mismatch repair-deficient gastrointestinal tumorigenesis and progression.. PLoS Genet.

