# Supplementary figures and images for: Genetic Mechanisms in Apc-Mediated Mammary Tumorigenesis
Source: PLoS Genet. 2009 Feb 6;5(2):e1000367. doi: 10.1371/journal.pgen.1000367 (PMC2629572; doi:10.1371/journal.pgen.1000367)

## Slide 1
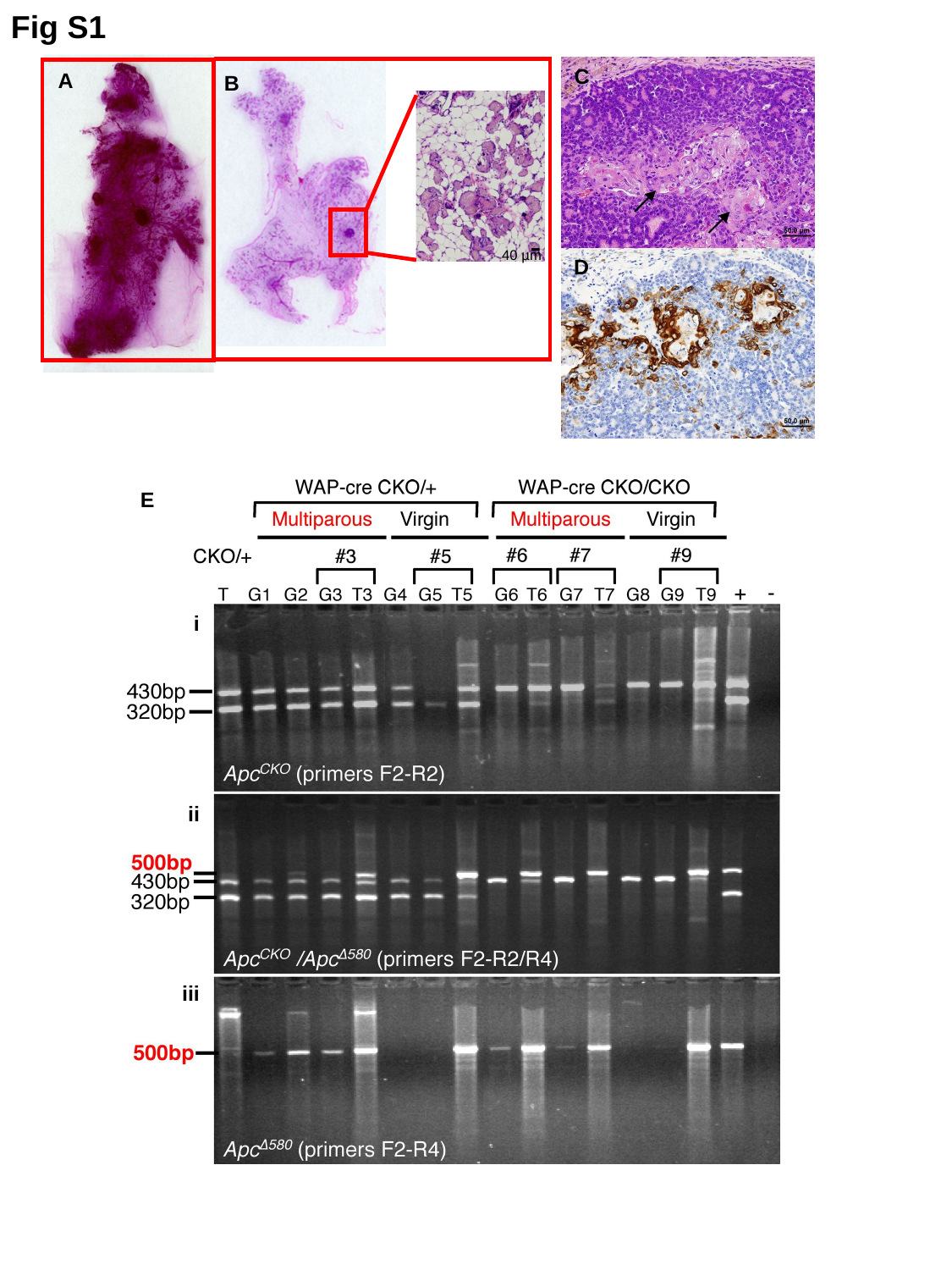

# Fig S1
A
B
40 µm
C
D
E
i
ii
iii

Supplement: Figure S1 — WAP-cre induced inactivation of Apc. (A) Whole-mount of a mammary gland showing development of severe squmaous metaplasia in multiparous WAP-cre; ApcCKO/CKO female mouse. (B) H&E staining of another gland from the same mouse showing multiple metaplstic lesions throughout the gland. (C) H&E staining of a mammary tumor from a multiparous WAP-cre; ApcCKO/CKO female mouse with squamous metaplasia (arrows) which have less defined structures than those in K14-cre; ApcCKO/+ tumors. (D) K14 expression was only observed in these metaplstic lesions in WAP-cre induced tumors. Scale bars: 50 µm. (E) Genotyping for Apc in WAP-cre positive mammary glands and tumors, showing WAP-cre and parity-specific recombination of conditional alleles. Genotyping PCR for the (i) wild-type and ApcCKO/+ alleles, (ii) wild-type, ApcCKO/+ and ApcΔ580 alleles, (iii) ApcΔ580 allele alone. The presence of the ApcΔ580 allele detected in mammary tumors from nulliparous WAP-cre positive females demonstrates that these tumors have derived from a clone of cells that have undergone Cre-mediated recombination. (3.0 MB PPT) [file pgen.1000367.s001.ppt]

## Slide 1
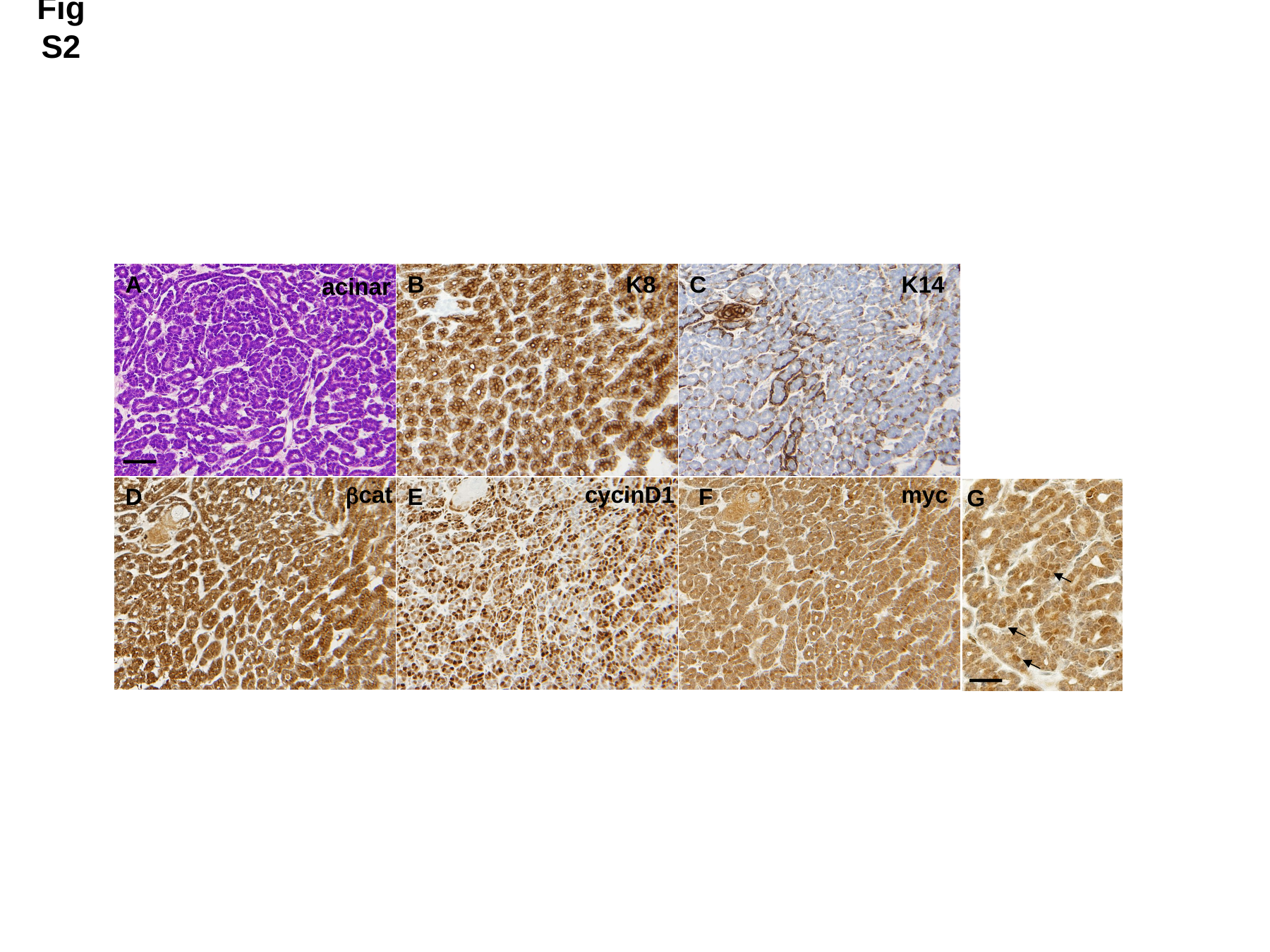

# Fig S2
A
acinar
B
K8
C
K14
βcat
cycinD1
myc
D
E
F
G

Supplement: Figure S2 — Expression of Tcf/β-catenin-target genes in mammary tumors from K14-cre; ApcCKO/+ female mice. A representative acinar-type mammary tumor stained with H&E for histology (A), luminal marker K8 (B), basal marker K14 (C) and β-catenin (D), showing strong positivity for Tcf/β-catenin-target genes cyclin D1 (E) and c-myc (F, G). Examples of cells with strong c-myc positivity are indicated by arrows (G). Scale bars: 50 µm for (A–F), 25 µm for (G). (7.8 MB PPT) [file pgen.1000367.s002.ppt]

## Slide 1
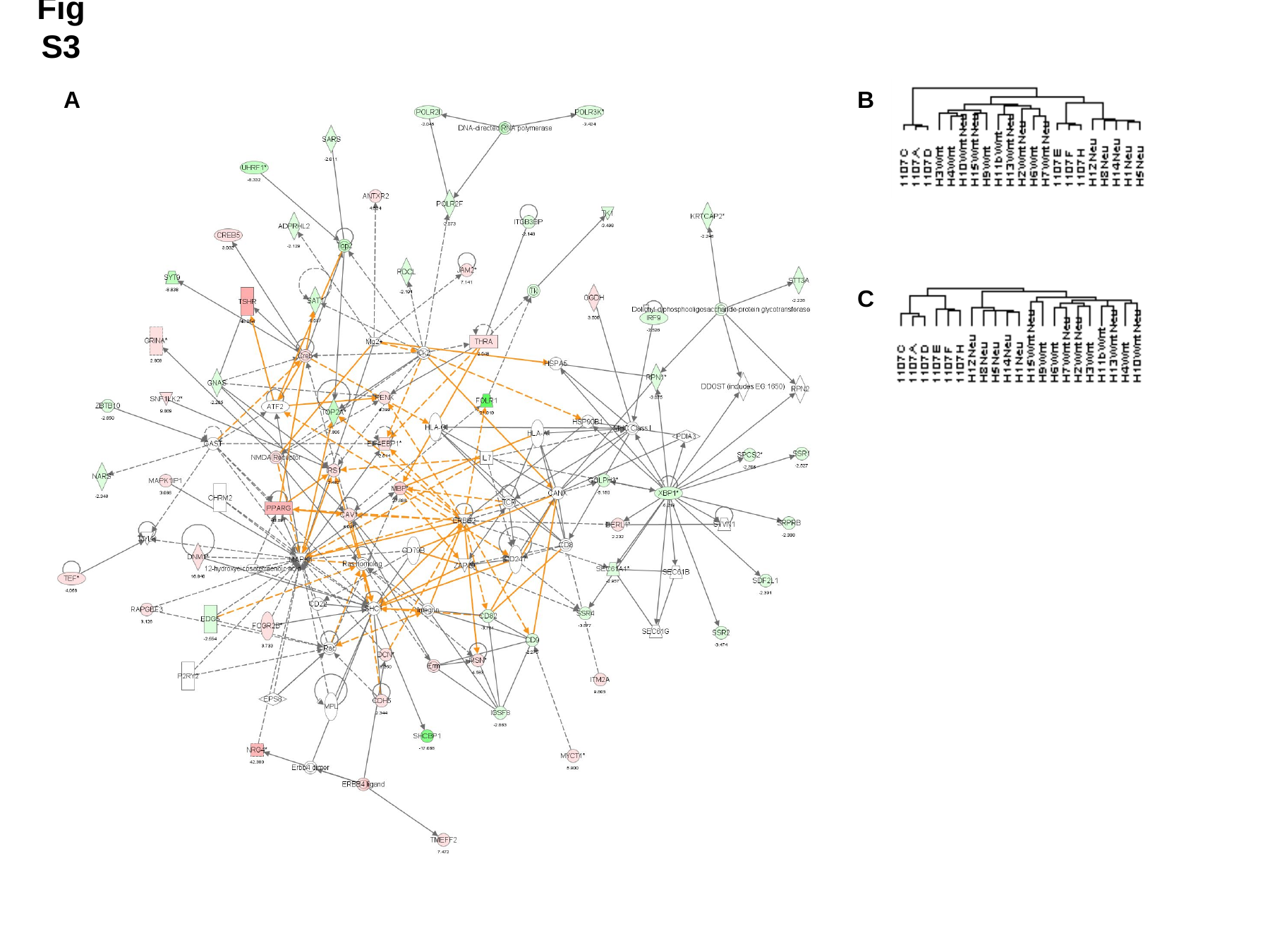

# Fig S3
A
B
C

Supplement: Figure S3 — Ingenuity Pathway and Hierarchical Clustering analyses of expression profiles of K14-cre; ApcCKO/+ mammary tumors. (A) A representative IPA network. Ingenuity core analysis generated over 60 networks, many of them involved in cell cycle, cell, growth, cell death, DNA replication and cancer (see Dataset S3 for information for all networks). A representative network is shown, showing elevated expression of Folr1. Green for elevated expression in tumor and red for elevation in control. (B) Hierarchical clustering by samples for 19,581 probes. A clustering diagram of K14-cre; ApcCKO/+ tumors (1107 E,F,H), controls (1107 A,C,D), tumors from MMTV-Wnt1, MMTV-Neu and MMTV-Wnt1/MMTV-Neu bitransgenic mice. Rank correlation was used as distance measure. (C) Hierarchical clustering by samples for 1,335 probes. 1,335 differentially expressed probes (fold differences above 2, t-test values above 4.5) from K14-cre; ApcCKO/+ tumors versus controls were used. (0.5 MB PPT) [file pgen.1000367.s003.ppt]
