# Supplementary material for: Genetic Mechanisms in Apc-Mediated Mammary Tumorigenesis
Source: PLoS Genet. 2009 Feb 6;5(2):e1000367. doi: 10.1371/journal.pgen.1000367 (PMC2629572; doi:10.1371/journal.pgen.1000367)
Supplement: Table S1 — Primer Sequences for Tp53 and Ras Mutational analysis. (0.05 MB DOC) [file pgen.1000367.s004.doc]

**Table S1 Primer Sequences for *Tp53* and *Ras* Mutational analysis**

| Primer | Prime | Use | Sequence |
| --- | --- | --- | --- |
| **For RT-PCR** |  |  |  |
| p53-F117 | 5’ | PCR | GTCTGTTATGTGCACGTACTC |
| p53-F121 | 5’ | Seq | GCACGTACTCTCCTCCCCT |
| p53-R308 | 3’ | Seq | AGGTGGGCAGCGCTCTCTT |
| p53-R313 | 3’ | PCR | GAGAGGCGCTTGTGCAGGT |
| Hras-Ex1F4 | 5’ | 1^st^ PCR/ Seq | GCAGTCGCGCCAGCAAGC |
| Hras-R100 | 3’ | 1^st^ PCR/ Seq | TGATCTGCTCCCTGTACTGAT |
| Hras-R190 | 3’ | 1^st^ PCR | ATGTCCTGAGCCTGGTGTCA |
| T3Hras-Ex1F4 | 5’ | 2^nd^ PCR | GCAATTAACCCTCACTAAAGGGCAGTCGCGCCAGCAAGC |
| T7Hras-R100 | 3’ | 2^nd^ PCR | AAGCTAATACGACTCACTATAGGGTGATCTGCTCCCTGTACTGAT |
| Kras-Ex1F1 | 5’ | 1^st^ PCR/ Seq | CCTGCTGAAAATGACTGAGTAT |
| Kras-R101 | 3’ | 1^st^ PCR/ Seq | TTAATTTGTTCTCTATAATGGTGAA |
| T3Kras-Ex1F1 | 5’ | 2^nd^ PCR | GCAATTAACCCTCACTAAAGGGCCTGCTGAAAATGACTGAGTAT |
| T7Kras-R101 | 3’ | 2^nd^ PCR | AAGCTAATACGACTCACTATAGGGTTAATTTGTTCTCTATAATGGTGAA |
|  |  |  |  |
| **For gDNA** |  |  |  |
| Hras-Ex1F1 | 5’ | PCR | CTTGGCTAAGTGTGCTTCTCA |
| Hras-Ex1R1 | 3’ | PCR | ACTGCCACAGCCCACCTCT |
| Hras-Ex1R2 | 3’ | Seq | CACCTCTGGCAGGTAGGCA |
| Kras-Int1F1 | 5’ | PCR | CACACAAAGGTGAGTGTTAAAAT |
| Kras-Int2R1 | 3’ | Seq | CTTTACAAGCGCACGCAGAC |
| Kras-Int2R2 | 3’ | PCR | CTGGCTGCCGTCCTTTACAA |
|  |  |  |  |

Use: PCR=for PCR, Seq= for sequencing
